# Biomarker discovery using machine learning in the psychosis spectrum

**DOI:** 10.1016/j.bionps.2024.100107

**Published:** 2024-08-26

**Authors:** Walid Yassin, Kendra M. Loedige, Cassandra M.J. Wannan, Kristina M. Holton, Jonathan Chevinsky, John Torous, Mei-Hua Hall, Rochelle Ruby Ye, Poornima Kumar, Sidhant Chopra, Kshitij Kumar, Jibran Y. Khokhar, Eric Margolis, Alessandro S. De Nadai

**Affiliations:** a Harvard Medical School, Boston, MA, USA; b Beth Israel Deaconess Medical Center, Boston, MA, USA; c Massachusetts General Hospital, Boston, MA, USA; d Western University, London, ON, Canada; e The University of Melbourne, Parkville, Victoria, Australia; f Orygen, Parkville, Victoria, Australia; g McLean Hospital, Belmont, MA, USA; h Yale University, New Haven, CT, USA; i Rutgers University, Piscataway, NJ, USA; j Harvard University, Boston, MA, USA

**Keywords:** Schizophrenia, Bipolar disorder, Psychosis, Machine learning, Clinical high risk, First episode

## Abstract

The past decade witnessed substantial discoveries related to the psychosis spectrum. Many of these discoveries resulted from pursuits of objective and quantifiable biomarkers in tandem with the application of analytical tools such as machine learning. These approaches provided exciting new insights that significantly helped improve precision in diagnosis, prognosis, and treatment. This article provides an overview of how machine learning has been employed in recent biomarker discovery research in the psychosis spectrum, which includes schizophrenia, schizoaffective disorders, bipolar disorder with psychosis, first episode psychosis, and clinical high risk for psychosis. It highlights both human and animal model studies and explores a varying range of the most impactful biomarkers including cognition, neuroimaging, electrophysiology, and digital markers. We specifically highlight new applications and opportunities for machine learning to impact noninvasive symptom monitoring, prediction of future diagnosis and treatment outcomes, integration of new methods with traditional clinical research and practice, and personalized medicine approaches.

## Introduction

The worldwide raw prevalence of the reported cases of schizophrenia ranges from 14.2 to 23.6 million, with disability-adjusted life years between 9.1 and 15.1 million (Solmi et al., 2023). In the US, schizophrenia and related psychotic spectrum disorders (PSD) prevalence is estimated to be between 0.25 % and 0.64 %, with an outstanding economic burden reaching $343.2 billion in 2019 for those with schizophrenia alone (Kadakia et al., 2022; Kessler et al., 2005; Wu et al., 2006). Psychotic experiences present in the general population are even 10 times more prevalent than established PSD which make up 10 % of the adult American population (Oh et al., 2018). The mortality rate of those with PSD after they are first diagnosed in their adolescence or young adulthood is 54.6 per 10,000 which is 8 times more than those who are typically developing, i.e., they die 10–15 years earlier, with some studies reporting shorter lifetime expectancy of about 15–20 years of life lost (Peritogiannis et al., 2022; Simon et al., 2018). These statistics are associated with several disorder-related factors including high physical morbidity linked to metabolic alterations and accelerated aging, in addition to others, such as sedentary lifestyle, smoking, and substance use (Peritogiannis et al., 2022). The psychosis spectrum, which includes established conditions such as schizophrenia, schizoaffective disorders (SAD), bipolar disorder with psychosis (BPD), as well as conditions such as first episode psychosis (FEP) and clinical high-risk for psychosis (CHR), manifests as varying levels of disruptions in perceptions, thoughts, and behaviors that could impede an individual’s daily functioning (American Psychiatric Association, and American Psychiatric Association, 2013). The categorization of PSD, like other neuropsychiatric conditions, suffers from the challenge of subjective assessment that contributes to its biological heterogeneity (Clementz et al., 2016; Lalousis et al., 2021; Voineskos et al., 2020). This heterogeneity constitutes a real challenge hampering assessment and treatment advancements (Clementz et al., 2016). Nonetheless, these challenges, among others, prompted a search for objective and quantifiable biomarkers, facilitated by analytical techniques like machine learning (ML), to enhance the accuracy of diagnosis, prognosis, and treatment. Among these markers, the most significant are cognition, neuroimaging, electrophysiology, and digital phenotyping. Cognition, including executive functioning, working memory (WM), and attention among others, has been reported to contribute significantly to the functional impairment of people on the psychosis spectrum (Bowie and Harvey, 2006; Fujiwara et al., 2015; McCutcheon et al., 2023). Neuroimaging, especially magnetic resonance imaging (MRI) witnessed substantial innovation over the past decade, increasing precision and overall data quality in both structural and functional measures which contributed to the identification of several brain regions involved in symptom onset, duration, and severity (Fujiwara et al., 2015; Howes et al., 2023; Keshavan et al., 2020; Voineskos et al., 2020). Electrophysiology, using electroencephalography (EEG), has a high temporal value, providing insight into the real-time dynamics of neural activity (Newson and Thiagarajan, 2019; Perrottelli et al., 2021). In addition, as individuals on the psychosis spectrum have perceptual aberrations, electrophysiological assessment tapping into indicators of perception including sensory gating can identify specific perceptual markers for targeted intervention (Chang et al., 2019; Ranlund et al., 2014). Furthermore, digital phenotyping can obtain a wide range of data including biometric data such as heart rate, and skin conductance, activity, social interaction, among others which are valuable in order to have a more comprehensive assessment (Cohen et al., 2023; Henson et al., 2020a).

ML has revolutionized the field of psychiatry, by providing several pragmatic solutions for prevailing challenges facing current diagnostic models such as heterogeneity (Clementz et al., 2016), comorbidity (Price et al., 2022), and one-size-fits-all therapeutic approaches (Wu et al., 2020). In addition, the ability to analyze large cohorts of a multivariate nature leads to robust investigations, identifying hidden patterns in the data, and rigorous evidence that informs discovery, care delivery, and enhanced outcome predictions. This can eventually help us efficiently and more accurately perform diagnosis, and prognosis, implement preventative techniques, and tailor treatment options, thus offering scalable solutions to address the growing demand for mental health services. Innovation in ML is occurring consistently and rapidly and has high promise of transforming psychiatric care delivery, offering hope for improved outcomes and better quality of life for all. In this review, we aim to delineate the impact ML has on the field of psychiatry, focusing on the psychosis spectrum in both human and animal model research through the most impactful biomarkers such as cognition, MRI, EEG, and digital markers.

## Machine learning and cognition

Cognitive deficits are considered to be a core feature of PSD. In individuals with chronic illness, deficits have been identified across virtually all cognitive domains, with the greatest levels of impairment observed in attention, executive function, speed of information processing, and memory (McCutcheon et al., 2023). The degree of impairment in individuals with chronic schizophrenia (chSCZ), is between one and two standard deviations below the mean of healthy controls (HCs) (Keefe et al., 2011), indicative of mild to moderate cognitive impairment. Similar deficits have also been observed in individuals with FEP. In those at CHR, more subtle cognitive impairments have been observed, with cognitive performance sitting approximately a quarter to half a standard deviation below normative means across multiple domains (Bolt et al., 2019). Furthermore, cognitive impairments are more pronounced in CHR individuals who later transition to frank psychosis, suggesting that they may have some predictive value in determining later clinical outcomes (Bolt et al., 2019).

Given the prevalence of cognitive deficits across all stages of PSD, it is unsurprising that measures of cognitive functioning have been examined as predictors of various outcomes in individuals experiencing psychosis. Key applications of cognition within ML frameworks include prediction of (1) diagnosis, (2) treatment response, (3) clinical outcomes, (4) functional outcomes, and (5) transition to frank psychosis. However, it is important to note that cognitive measures have typically been included alongside a range of other measures, including clinical, sociodemographic, and neuroimaging data, when developing prediction models in psychosis.

Several studies indicate that cognitive data have utility for the prediction of PSD versus HC. A recent study in individuals with antipsychotic-naive FEP, which utilized an ensemble of ML algorithms, found that the correct classification of diagnosis (i.e., FEP versus HC) in a multimodal ML framework was driven by cognitive data (Ambrosen et al., 2020). Importantly, while the overall balanced accuracy (BAC) of the model was only 64.2 % when all variables were included, classification accuracy based only on cognitive variables resulted in a BAC of 67.8 %. In another study of individuals with early psychosis, the classification accuracy of a deep learning framework utilizing structural neuroimaging data was improved with the addition of cognitive estimation as a subtask (AUC 71.1 ± 4.1) (Wen et al., 2023). In individuals with early-onset FEP, support vector machine (SVM) classification of diagnosis using jackknifing resulted in an accurate classification of 87 % (sensitivity 0.85; specificity 0.88) (Pina-Camacho et al., 2015). In this study, impaired attention, motor coordination, and global cognition were the strongest predictors of early onset FEP (Pina-Camacho et al., 2015). In individuals with multi-episode schizophrenia, raw cognitive scores were predictive of the schizophrenia diagnosis versus HC with AUC ranging from 0.67 for processing speed to 0.86 and 0.87 for verbal memory delayed and immediate recall scores, respectively (Gómez-gastiasoro et al., 2020). Similarly, Doan et al. (2017), found that cognitive scores, both on their own and combined with neuroimaging and polygenic risk scores, were able to predict schizophrenia versus HC with relatively good accuracy (AUC 0.81–0.85) when using random forest algorithms (Doan et al., 2017).

Cognitive data has also been shown to be useful in predicting diagnosis in PSD versus non-PSD, although findings are equivocal. In early onset FEP, cognitive variables alone were not able to accurately predict FEP versus non-FEP (accuracy 63 %), despite multimodal prediction models in the same sample achieving diagnostic accuracy of almost 80 % (Pina-Camacho et al., 2015). However, a study of individuals with FEP was able to correctly classify 80.81 % of first episode patients (versus depression and HC) using a three-class AdaBoost tree-based ensemble algorithm. In this study, cognitive measures that best separated individuals with FEP from those with depression included motor speed, general intelligence, perceptual sensitivity, and reversal learning. Recent studies have also sought to predict schizophrenia versus BPD using cognitive variables. Fernandes et al. were able to predict schizophrenia versus BPD using cognitive variables with an AUC of 0.77 (Doan et al., 2017; Fernandes et al., 2020). Cognitive domains with the strongest contribution to prediction included verbal learning, WM, visuospatial ability, and premorbid intelligence quotient (IQ). Similarly, Doan et al. (2017), used random forest to investigate the predictive accuracy of neuroimaging features alone, cognition alone, and combined neuroimaging and cognition measures to predict schizophrenia versus BPD. However, they found that neither cognitive measures alone nor combined neuroimaging and cognitive measures were able to differentiate schizophrenia and BPD with sufficient accuracy (AUC 0.62 and 0.61, respectively).

There is widespread impairment of cognitive ability in individuals at CHR compared to HC, impacting around 45.9 % of the population (Haining et al., 2022). Cognitive impairments are evidenced in all neurocognitive domains to varying degrees, with impairment observed particularly in processing speed, attention, WM, and, reasoning (W. Zheng et al., 2018), Meta-analytic studies show evidence of a cognitive decline in patients with CHR and FEP, suggesting that cognitive deficits are already established before onset of psychosis (Bora and Murray, 2014). Additionally, individuals at CHR who transition to FEP show larger cognitive impairments compared to individuals who do not transition, suggesting that cognitive impairments may be useful in differentiating transition trajectories from CHR (Carrión et al., 2015). Research shows that baseline cognitive deficits in verbal learning, visual memory, processing speed, and attention are associated with a longitudinal risk of psychosis onset (Catalan et al., 2021). These cognitive impairments may be candidates for unimodal prediction models utilizing cognitive data, or for refining multivariable prediction models that integrate multimodal data (E.g., clinical, cognitive, and neuroimaging data) (Catalan et al., 2021).

Cognitive data have been successful in differentiating CHR from HC. A study investigating the diagnostic classification of CHR and HC achieved a BAC of 87.9 % using a comprehensive neuropsychological test battery (Koutsouleris et al., 2012). The investigators found the highest feature selection probabilities and reliable group differences observed for premorbid verbal IQ, cognitive set-shifting, visual WM, and verbal learning. They also demonstrated that compared to HC, both CHR transitions and non-transitions exhibited significant performance deficits across multiple cognitive domains.

In predicting CHR transition to frank psychosis, the cognitive model developed by Koutsouleris et al. (2012), was able to predict CHR transition with an impressive BAC of 93.3 %, and non-transition with a slightly lower BAC of 86.7 %. The model was further able to distinguish CHR transition versus non-transition with a BAC of 77.5 %. This distinction pattern involved verbal and executive functions, such as cognitive flexibility and verbal learning (Koutsouleris et al., 2012). The researchers note that larger training samples and additional neurocognitive information from a HC group facilitate the modeling of the complex discrimination boundary between CHR transition versus non-transition, evident in the lower BAC when the HC group is removed. One study found low baseline IQ to be the only significant neurocognitive predictor of transition to FEP (AUC 0.77), although another study showed supporting evidence for the predictive ability of processing speed and verbal IQ, highlighting some heterogeneity regarding the specific domains implicated in the prediction of transition (Riecher-Rössler et al., 2009; Ziermans et al., 2014). In both aforementioned studies, combining cognitive and clinical data produced a more accurate model. Ziermans et al. (2014), found that a multimodal approach improved the AUC from 0.77 to 0.82, whereas Riecher-Rossler et al., 2009, found a multimodal clinical-cognitive model performed better (BAC 81.3 % versus BAC 69.3 %), specifically with the inclusion of processing speed in the clinical model. As such, findings appear to favor the integration of neurocognitive data into clinical models in order to improve predictive ability, rather than unimodal cognitive models.

In FEP, cognitive ability has typically been examined as a predictor of functional and clinical outcomes. Memory, verbal fluency, and social cognition have been identified as predictors of remission and relapse in FEP (Schubert et al., 2015). However, while studies have identified specific cognitive domains to be significant predictors of illness course or functional outcomes in FEP, the amount of variance explained by these cognitive measures is very small and they are therefore insufficient on their own to predict FEP outcomes (Schubert et al., 2015). A recent multimodal ML study, which included cognitive measures, was unable to predict either short- or long-term treatment response in FEP individuals (Ambrosen et al., 2020). Additionally, a study using a decision tree algorithm found that cognitive measures were not strong predictors of psychotic relapse at 2 years post psychosis onset. Together, these findings suggest that cognitive scores may not be key predictors of illness trajectories or outcomes in FEP individuals (Fond et al., 2019).

In CHR, aside from clinical outcomes (i.e., transition to frank psychosis), cognitive ability has also been used to assess long-term functional outcome. A 13-year longitudinal study of individuals at CHR revealed that poor functional outcome at follow-up was predicted by baseline performance in verbal learning and memory, verbal fluency, and to a lesser extent by processing speed and attention (Lin et al., 2011). The study found that lower performance in logical memory at baseline was the strongest predictor of poor functional outcome at follow-up. A more recent study highlighted that poor verbal memory at baseline predicted non-remission and greater functional disability at two-year follow-up (Hedges et al., 2022). However, when considered with clinical and functioning variables, cognitive ability appears to be no better predictor than baseline functioning or clinical symptoms, such as negative symptoms (Brandizzi et al., 2015; Glenthøj et al., 2020). Similarly, another study employing a range of ML algorithms, including SVM, linear discrimination analysis (LDA), and random forest classification, found that global functioning scores at baseline alone predicted social and role functional outcomes in CHR at follow-up better than multimodal models combining clinical, cognitive, and functioning variables (BAC 68 % versus BAC 63 %) (Haining et al., 2021). Therefore, while cognitive ability at baseline is predictive of functional outcomes in CHR populations, it does not appear to be a better method of prediction than clinical and functional measures. It appears that clinical and functional measures are just as, if not more, predictive than cognitive ability alone.

Numerous studies have examined cognition as a predictor of later functioning in individuals experiencing FEP. A meta-analysis of these studies from 2017 found that multiple cognitive domains, including general cognitive ability, attention, WM, verbal fluency, verbal learning and memory, executive functioning, processing speed, non- verbal learning and memory, and visuomotor skills were all significant predictors of later functional outcomes in FEP individuals (Santesteban-Echarri et al., 2017). More recently, a study examining cognition as a predictor of real-world functioning in individuals with schizophrenia found that a cognition composite was a significant predictor of functioning, explaining 11 % of the variance in functioning at follow-up (Yang et al., 2021). Similarly, processing speed was associated with 1-year remission, occupational status, and maintaining of life goals in individuals experiencing FEP, although this prediction was no longer significant when negative symptoms were included in the model (Lindgren et al., 2020). A recent study using a bagging ensemble framework to predict functional outcomes in schizophrenia found that a combination of category fluency, Wechsler Abbreviated Scale Intelligence (WAIS)-III digit symbol- coding, verbal WM, nonverbal WM, and social cognition best predicted quality of life scores, whereas a combination of category fluency, WAIS-III digit symbol-coding, d- Prime of blurred version, verbal WM, and reasoning and problem-solving best predicted global functioning (Lin et al., 2021). However, prediction of functioning using cognitive measures was not able to surpass prediction performance when clinical variables were used.

## Machine learning and magnetic resonance imaging

Neuroimaging research yielded crucial insights into the pathophysiology of PSD. Structural and functional aberrations, including impaired myelination, progressive degeneration of cortical thickness (CT) and gray matter volume (GMV), and altered functional connectivity (FC) between widespread brain regions, have been consistently observed in individuals across the PSD. These findings served as the basis for employing ML techniques in pursuit of developing reliable tools for differentiating individuals on the PSD from HC and other patient groups, as well as identifying biologically homogeneous subgroups and predicting treatment outcomes.

A recent meta-analysis that included 28 structural neuroimaging, 9 diffusion tensor imaging (DTI), 38 resting-state FC (rsFC), and 14 multimodal studies reported that neuroimaging data have substantial utility in distinguishing individuals on the psychosis spectrum from HC, with similar classification accuracy for all imaging modalities (Porter et al., 2023). The rsFC had a slight advantage over structural connectivity metrics (Porter et al., 2023). Other studies highlight the importance of differentiating between different diagnostic groups, especially in those with potential overlap. A recent study (Yassin et al., 2020), showed that classifiers trained on multiple structural neuroimaging metrics (CT, surface area, and subcortical volume) distinguished individuals with schizophrenia from those with ASD (85 %) and HC (76 %) with the highest accuracy using subcortical volume. The authors also ran a multiclass classifier on all three groups that best distinguished them using CT with a 70 % accuracy (Yassin et al., 2020).

A large multi-site study that included data from 8 centers showed that vertex-wise thickness outperformed parcellation-based methods in classifying FEP versus HC (66.2 % accuracy), with temporal brain areas being the most influential in the classification (Pigoni et al., 2021). Another study applied the model that was trained on GMV to differentiate chSCZ from HC (test accuracy 75 %, independent dataset accuracy, 76 %) from ASD, FEP and, CHR (Y. Zhu et al., 2022). Those on the psychosis spectrum were more likely to be classified as chSZ compared to ASD (classification rate to chSCZ: CHR, 41 %; FEP, 54 %; ChSZ, 70 %; ASD, 19 %; HC, 21 %). Using a similar approach, Yassin and colleagues used classifiers trained to differentiate schizophrenia, HC and, ASD, and showed that CHR and FEP participants were mostly classified as schizophrenia (55 % and 70 % respectively, with the rest as HC, but critically, none as ASD), indicating that those at CHR have brain patterns similar to those with schizophrenia early in the course of illness (Yassin et al., 2020). The variability in performance accuracy across these studies could be attributed to the use of different structural metrics and multi-site data bringing in the heterogeneity of the samples and data acquisition protocols (Pigoni et al., 2021; Zhu et al., 2022). Interestingly, Pigoni et al. showed that by including scanner-specific variables and demographics in the model, the accuracy increased by 4 %, improving the across-site generalizability (Pigoni et al., 2021). Complementing this, a recent study found that by harmonizing measures of structural metrics from across different sites and correcting for non-linear effects of age and sex, the XGBoost classifier achieved an 85 % accuracy on the training set and 73 % accuracy on an independent confirmatory dataset in classifying individuals with CHR who transitioned to psychosis (CHR-PS+) from HC (Zhu et al., 2023). The key neuroanatomical features that contributed to the classifier’s predictive ability were surface area measures from regions such as the superior temporal, insula, superior frontal, fusiform, and parahippocampal cortices – areas that have been previously implicated in psychosis and schizophrenia. Importantly, when the trained classifier was applied to individuals at CHR who did not develop psychosis (CHR-PS−) or had an unknown outcome, it was more likely to classify them as HC compared to the CHR-PS+ group. These results further indicate that neuroanatomical deviations were already present at baseline in those who later transitioned.

Resting state FC metrics have also been shown to distinguish PSD effectively (Rashid et al., 2016). One large-scale study reported that whole brain dynamic FC achieved 69 % performance accuracy in distinguishing between four groups, schizophrenia, SAD, BPD and HC and more than 80 % mean overall accuracy in between-group binary classifications (Du et al., 2020). A high level of classification between BPD and schizophrenia was achieved using effective connectivity and morphometry of the triple network (default mode network (DMN), fronto-parietal network, and salience network) (Palaniyappan et al., 2019).

A recent study (Du et al., 2022), showed that adopting a fused classifier significantly improved the classification performance (test (83 %), independent dataset (72 %)), exceeding single-modality features. Specifically, the authors combined the classifiers’ outputs from different modalities (structural GMV, and FC metrics) using a linear weighting method to generate the final predicted label. This study revealed the most important features that contributed to the classification of schizophrenia versus ASD and HC, including the subcortical, DMN, and visual network FCs. In contrast, structural GMV group differences were focused on the frontal and temporal gyri and right insula. However, conducting classification at the features level and not just the model level would further improve the classification accuracy of different disorders, as combining data from different modalities before the statistical stage will improve the power by leveraging the covariance structure in the multi-modal data within each subject (Gong et al., 2023; Groves et al., 2011).

Individuals across the psychosis spectrum show substantial heterogeneity in brain and behavioral phenotypes, including within discrete diagnostic categories such as schizophrenia (Barch et al., 2013; Wolfers et al., 2018). To parse this heterogeneity, a common approach has been to use unsupervised or semi-supervised clustering algorithms on structural and functional MRI derivatives from large groups of clinically or phenotypically homogeneous individuals to identify biologically homogeneous clusters— an approach that has shown success in identifying validated subgroup biomarkers in psychosis (Clementz et al., 2016, 2022; Voineskos et al., 2020). Clustering approaches applied to structural MRI derivatives such as GMV or CT (Pan et al., 2020; Xiao et al., 2022) have reliably demonstrated two neuroanatomical subtypes in PSD (Chand et al., 2020; Dwyer et al., 2018; Lalousis et al., 2022; Planchuelo-Gómez et al., 2020; Shi et al., 2023): 1) individuals with widespread reduction in GMV, particularly in subcortex and higher-order cortical association areas, and 2) individuals with relatively preserved neuroanatomy with possibly increased volume in basal ganglia. Although other numbers of clusters have also been reported (Honnorat et al., 2019; Pan et al., 2020; Xiao et al., 2022). Generally, these ‘bottom-up’ ML approaches have identified neuroimaging-based biomarkers representing individuals with and without neural deficits and intermediate subgroups representing mixtures of these two groups. Neuroanatomical subtypes showing widespread reductions in GMV have generally been shown to be linked to longer illness duration and more severe symptoms (Chand et al., 2020; Dwyer et al., 2018; Lalousis et al., 2022; Planchuelo-Gómez et al., 2020; Shi et al., 2023).

Other neuroimaging-based ML subtyping approaches take into account the temporal progression of neuroanatomical changes often reported in PSD (Jiang, et al., 2023; Jiang, Wang, et al., 2023). One added advantage of these methods is the identification of ‘epicenter’ biomarkers that index not only the observed neuroanatomical aberrations but also the brain region of initial pathological onset (Young et al., 2018). This approach has again identified two subtypes in established schizophrenia (Jiang, et al., 2023; Jiang, Wang, et al., 2023), that initiate from either frontal or hippocampal epicenters, with the former resulting in greater cortical gray matter loss and better antipsychotic response (Jiang, et al., 2023).

A commonly reported neural aberration across the psychosis spectrum can be found in FC. However, the spatial pattern of the reported aberrations is highly heterogeneous, potentially suggesting that distinct patterns of FC may define subgroups of patients.

One study applied unsupervised clustering to FC from three higher-order networks in 300 antipsychotic-naive FEP individuals (Liang et al., 2021). They found two subgroups that were both validated longitudinally in an independent sample. Their findings further indicated that one subgroup was defined by severe clinical symptoms and salience network hypoconnectivity and the other exhibited widespread hyperconnectivity and greater deficits in neurocognition (Liang et al., 2021). These subgroups align with decades of research demonstrating the presence of a ‘deficit’ subtype of patients with high negative symptoms and cognitive aberrations (Kirkpatrick and Galderisi, 2008).

Indeed, more explicit attempts to distinguish subgroups with such deficits using a combination of structural and functional MRI have achieved a high accuracy (75.48 %) in identifying these individuals among patient samples (Gao et al., 2023), but has not been validated in independent samples (Gao et al., 2023).

FC has also been utilized to validate clusters defined using other modalities. In one of the most rigorous subtyping studies to date, four externally cross-validated and longitudinally stable clusters were defined using Non-Negative Matrix Factorization applied to common measures of symptom severity in 1545 individuals with schizophrenia (Chen et al., 2020). While these clusters were defined using symptoms, they could also be successfully classified using SVM applied to FC data from patients, suggesting a biological basis for these behaviorally defined subgroups.

In contrast to the majority of robust subtyping approaches, promising and validated MRI-based biomarkers of treatment response have come from relatively simple theory- driven applications of ML to functional MRI data. In particular, striatal functioning –a primary target of antipsychotic medications–has provided the most effective and reliable results to date for prospectively predicting antipsychotic responses. Two markers, in particular, have been validated using independent samples, with the first (Striatal Connectivity Index (Sarpal et al., 2016)); using a simple measure of FC between the striatum and rest of the brain, achieving 80 % sensitivity and 75 % specificity in predicting a dichotomous measure of antipsychotic response in a replication cohort of 40 patients (Sarpal et al., 2016). This finding is consistent with a recent meta-analysis of predicting antipsychotic response using functional connectivity, demonstrating a sensitivity of 81 % and specificity of 76 % (Mehta et al., 2021). The second (Functional Striatal Abnormality score (Li et al., 2020)) uses an SVM to combine features of regional striatal functioning and intra- and extra-striatal functional connectivity to generate a subject-level score that reliably predicts both diagnostic status and a continuous measure of antipsychotic response in patients with established schizophrenia, achieving significant correlations of r=.62 (n=37) and r=.42 (n=58) in two independent cohorts of patients assessed longitudinally over 6-weeks (Li et al., 2020).

Building on the success of functional MRI prediction of treatment response, other studies have taken a more data-driven and whole-brain approach to predicting antipsychotic response using FC. One popular and promising framework called connectome-based predictive modeling (Shen et al., 2017), uses whole-brain region-to- region connectivity matrices, selecting brain features most correlated to the outcome and then taking the mean or sum of selected features as input into any cross-validated prediction model. This framework has shown success in prospectively predicting longitudinal changes in a broad range of phenotypes using functional (Cao et al., 2023), and diffusion-weighted imaging (Chopra et al., 2023; Sun et al., 2022), in psychosis. It was also recently validated to predict a continuous measure of treatment outcomes using baseline whole-brain functional connectivity across 12 weeks at r=.47 in an independent validation sample of 24 FEP patients (Cao et al., 2023).

Recent advancements in ML have led to the development of normative models that chart the typical development of brain phenotypes across the lifespan using ultra-large samples (Ge et al., 2024). These models enable the computation of patient-level regional deviation scores, indicating the level of aberration in an individual’s brain phenotype compared to a large normative population, accounting for critical variables such as age and sex. Individualized measures of deviation have demonstrated more robust and stronger associations with continuous measures of treatment response compared to raw measures of brain volume in a sample of FEP subjects, particularly in striatal regions (Remiszewski et al., 2022), but are yet to be validated in independent cohorts of patients.

While ML methods applied to MRI data to predict antipsychotic treatment response have revealed several validated markers that generalize between independent samples, further work is still required to demonstrate clinical utility, such as efficacy and effectiveness in clinical settings. Finally, an important barrier to overcome is the lack of consensus on measures of treatment response and, relatedly, a focus on symptoms rather than functioning, such as social and occupational outcomes. Field consensus on prediction targets of treatment response shaped by patient preferences and lived experience involvement will likely be needed to achieve clinical utility for MRI-based biomarkers.

These findings demonstrate the potential of ML approaches, when applied to multisite neuroimaging data, to identify individuals at CHR based on their pre-onset brain patterns. By leveraging the predictive power of ML models, clinicians could potentially identify those CHR individuals most in need of preventive interventions or closer monitoring, thereby improving treatment efficacy and outcomes.

## Machine learning and EEG

EEG and event-related potential (ERP) methods are non-invasive tools with superior temporal precision, offering insights into the dynamics of brain activity associated with both cognitive functions and impairments among other deficits. These techniques allow for the discovery of neurophysiological biomarkers and the exploration of the neurobiological underpinnings of psychiatric conditions.

PSD can especially benefit from EEG and ERP for biomarker identification as several related biomarkers have been identified to be altered, such as mismatch negativity (MMN), sensory gating/P50, P300, N100, gamma band oscillations (30–80 Hz), and the auditory steady-state response (ASSR) (Barros et al., 2021; Javitt et al., 2008). The P300 (Donchin et al., 1978), reflects attention and memory function, P50 (Javitt and Freedman, 2015; Potter et al., 2005), and N100 (Freedman, 2014) are associated with sensory processing, whereas MMN (Näätänen et al., 2016) indicates the perceptual experience of pre-attentive deviance detection. These markers are robustly impaired in individuals with schizophrenia and other PSD, pointing to cognitive, sensory, and perceptual deficits ^61,62–67^. Furthermore, abnormalities in gamma band oscillations and ASSR are indicative of disrupted neural connectivity/communication and have been associated with primary psychosis symptoms, such as hallucinations and delusions (Zhou et al., 2018; Thuné et al., 2016). Using EEG and ERP as tools to study neurocognitive functions greatly enhances our understanding of patient variability within psychosis, and allows for its prediction (Lee and Kim, 2022; Ramyead et al., 2016).

ML techniques hold significant promise for advancing the analysis of EEG and ERP biomarker data, presenting powerful tools to decode the neural complexity of psychiatric disorders. These computational approaches can be particularly adept at handling the high-dimensional, non-linear, and noisy nature of neurophysiological data, making them optimal for extracting meaningful patterns and biomarkers from EEG and ERP signals (Saeidi et al., 2021). Within the context of psychosis, the application of ML to EEG data offers the potential to: a) confirm diagnoses; b) predict responses to treatments or interventions; c) classify disease progression and outcomes; and d) elucidate the neurocognitive mechanisms underlying clinical symptoms, as noted by Barros et al. (Barros et al., 2021).

ML has been applied to EEG and ERP in an array of psychiatric disorders for various classification purposes. In the psychosis spectrum, for instance, Shim et al. employed ML algorithms to analyze ERP components, classifying patients with schizophrenia versus HC, and predicting medication responses (Shim et al., 2016). Olbrich and Arns reviewed ML methods to EEG data to predict the efficacy of antidepressant treatment in major depressive disorder (MDD) (Olbrich and Arns, 2013). In another article, Del Fabro et al. reviewed ML studies using multiple modalities including EEG and ERP features to predict antipsychotic treatment response in individuals with early and chSCZ. This work emphasizes the heterogeneous nature of using multiple modalities like EEG, structural MRI, functional MRI, and clinical covariates as predictors, and the potential of ML in handling complex, high-dimensional data (Del Fabro et al., 2023). Another study by de Nijs et al. applied ML for symptomatic outcome and long-term global outcome (via global assessment of functioning) prediction in schizophrenia-spectrum patients, with 62.2–64.7 % accuracy in symptomatic outcome, and 63.5–67.6 % accuracy in global outcome(de Nijs et al., 2021). Ramyead et al. employed ML in analyzing patterns of beta and gamma oscillations to evaluate if aberrant patterns preceded and predicted psychosis in neuroleptic-naïve, CHR subjects, with current-source density EEG achieving a predictive AUC of 0.77 (Ramyead et al., 2016). Utilizing ML with the gamma band of P50 components, Luo et al. classified the progression of psychosis based on brain connectivity, in CHR, and FEP, to distinguish between the subtypes and aid in early diagnosis (Luo et al., 2019). Similarly, Chang et al. applied ML to P50 sensory gating to find differences in brain connectivity features between CHR and HC, and FEP and HC, to aid diagnosis and early intervention (Chang et al., 2019).

Looking at the different algorithm solutions applied to EEG, Barros et al. reviewed 26 psychosis-related EEG studies, with a focus on their methodology. SVM was the most commonly applied classical ML algorithm, followed by LDA (Barros et al., 2021).

Stepping into the realm of deep learning, convolutional neural networks (CNN) were the most frequently utilized model in psychosis-related EEG studies (Barros et al., 2021).

Indeed, Ahmedt-Aristizabal et al. found that CNN outperformed classical ML on the EEG waveforms from the passive auditory oddball paradigm in children at risk for schizophrenia (Ahmedt-Aristizabal et al., 2021). Similarly, Ravan et al. coupled brain source localization of EEG decomposed by frequency with CNN to classify MDD, MDD-Atypical, MDD-Psychotic, Bipolar-Depressive, Bipolar-Manic, and schizophrenia from HC with over 90 % accuracy (Ravan et al., 2024). These studies collectively underscore the significant impact of ML on psychiatric research, especially the psychosis spectrum, through the lens of EEG and ERP data. By elucidating the complexity of EEG and ERP signals, ML approaches are paving the way for enhanced diagnostic precision, tailored therapeutic interventions, and a deeper comprehension of the biological underpinnings of mental health conditions.

To our knowledge, no study has yet implemented ML on the ASSR. While 40 Hz ASSR deficits have been consistently observed in patients with psychosis (Thuné et al., 2016; Onitsuka et al., 2022), and studies have used sensor level analyses for pinpointing the ASSR deficits, the majority of studies have been limited to analyzing a select number of channels, typically focusing on the fronto-central channels of the EEG (Onitsuka et al., 2022). This focus tends to overlook the discriminative potential of channels across the scalp, indicating areas where the signal to noise ratio can best be observed for use in classification. Consequently, the exact, detailed patterns of deficits in individual cases available in high density (e.g., 64 or 128 electrodes), EEGs are often ignored. A plethora of ML methods can handle multivariate and complex data and extract or select representations of relevant data features for training classifiers (Saeidi et al., 2021). The accuracy of the classification depends on the choice of the algorithm for the type of data at hand. Administering an ensemble of classifiers in both ASSR paradigms of evoked power (PWE) and phase-locking factor (PLF) can reveal which type of classifier performs the best with respect to each paradigm, while permutation analysis can boost the robustness of the classifiers. With some types of classifiers, the importance of each channel in either PLF or PWE can be assessed directly, but for other classifiers, the channel importance is assessed by examining the classification accuracy with and without that channel in the model. Taking this idea further, one can create ensemble rankings of each channel for PLF and PWE across the different classifiers revealing highly discriminatory channels. Through this ML approach, we can assess EEG patterns in whole-scalp 40 Hz ASSR as potential biomarkers of psychosis. To date, we are unaware of studies of ASSR applying ML methods on EEG with whole-scalp electrodes, and therefore, we are currently carrying out a study to address this gap.

ML techniques offer transformative tools for refining and extracting valuable insights from EEG and ERP data in the study of psychosis. By harnessing the power of ML algorithms, researchers can uncover complex neural patterns for personalized treatment strategies. The application of ML to 40 Hz ASSR studies presents a particularly promising avenue for investigation. Further research into utilizing ML on the scalp-level multi-channel ASSR has the potential to unlock novel biomarkers for psychosis, leading to the development of personalized treatment

## Machine learning and digital markers

Digital interventions for use in the screening and treatment of PSD include a wide range of existent technologies that take advantage of digital biomarkers. The Food and Drug Administration defines a digital Biomarker as a “characteristic or set of characteristics, collected from digital health technologies, that is measured as an indicator of normal biological processes, pathogenic processes, or responses to an exposure or intervention, including therapeutic interventions.” (Javitt et al., 2008; Vasudevan et al., 2022) Such digital health technologies (DHT) include several sources of data, ranging from software to physical sensors to hardware, such as computing platforms.

Many DHTs will meet the definition of a device in accordance with the Federal Food, Drug, and Cosmetic Act (Commissioner, n.d.). As such, prior to clearance, they would require verification, validation, and evaluation of usability.

There are a few common DHTs that have been utilized for establishing digital biomarkers for PSD which warrant discussion here. Ecological momentary assessments (EMA) involve serial assessments of an individual’s current experiences and can be accomplished through survey data. EMAs have been used in a multi-center study of FEP utilizing a phone application and have been shown to be feasible and acceptable, with similar efficacy in cognitive and clinical parameters as established tools (Lakhtakia et al., 2022). Wearable technologies have also been used to collect biological data in a similar manner, with studies showing close acceptability and reliability of such technologies in tracking autonomic abnormalities in individuals with schizophrenia (Cella et al., 2018). Digital phenotyping is a strategy that incorporates both EMA survey data and wearable technology data into a combined data pool to enhance the accuracy and generalizability of future predictions. The use of the combination of such passive and active data sources in screening for anomalous behavior has been shown to have some predictive utility in screening for relapse in individuals with schizophrenia (Cohen et al., 2023).

ML, given its potential for establishing trends in large datasets, becomes a tantalizing tool for the evaluation of digital phenotyping information in PSD. Unsurprisingly, many researchers have utilized different ML approaches to attempt just that. However, there is no established consensus on how to implement this approach as of yet. In a systematic review of the use of ML for digital phenotyping data in PSD, 16 studies using ML techniques were found, including techniques such as neural networks, random forest, and SVM, but generalizability was significantly limited by small sample sizes (average of 23 participants per study) (Benoit et al., 2020a).

However, some potentially useful biomarkers have been investigated in the sphere of digital phenotyping for PSD. In schizophrenia, for example, the relapse and severity of positive symptoms, have been found to be negatively associated with the quantity of outgoing text messages and the duration of phone calls and positively associated with the total amount of time spent at one’s primary location (Buck et al., 2019, 2022). As for negative symptoms, accelerometer and GPS data have unsurprisingly demonstrated an association with decreased mobility and less time spent outside of the home with worsening symptoms (Depp et al., 2019). Sensor data has also been used to track the degree of stability of routines, with higher stability correlating with lower rates of both positive and negative symptoms of schizophrenia (Henson et al., 2020b).

Sleep has also been investigated in this population using digital phenotypic techniques, with studies showing that poorer sleep quality, in individuals with schizophrenia, is associated with poorer treatment adherence (Afonso et al., 2014), and could be used retrospectively to predict psychotic symptoms in the following weeks (Meyer et al., 2022). Finally, predicting the relapse of psychotic symptoms has been a major target of research efforts. Similar to positive symptoms, relapse has been shown to be negatively associated with the quantity of outgoing text messages and the duration of phone calls (Buck et al., 2019). Despite this, no factor has been shown to be as predictive of relapse as medication non-adherence (Di Capite et al., 2018). Therefore, interventions such as Abilify MyCite (Otsuka America Pharmaceutical, Inc. n.d.), aripiprazole tablets with a built-in sensor to monitor adherence, may represent a movement towards digital biomarkers with high predictive value.

Still, validation into diverse populations and disparate data collection methodologies remain significant limitations (Zarate et al., 2022). The lack of standardization of data collection and ML protocols in all psychiatric disorders will remain a barrier to the reproducibility of such research for the foreseeable future. Such limitations are even more pronounced in the lesser-studied psychosis spectrum. Many individuals with PSD are screened out from prospective digital phenotyping studies; moreover, those studies that are focused on PSD prevention and treatment likely suffer from selection bias, as individuals with more severe psychotic symptomatology, cognitive impairment, or refractory condition would likely be excluded from participation in such studies.

There are some additional challenges to utilizing technology and digital phenotypic approaches in individuals with PSD. The ethical considerations surrounding informed consent for participation are paramount. Many individuals with PSD may lack the capacity to provide informed consent for involvement in research studies; and if they are involved in such studies, cognitive limitations may lead to poor engagement with or limited utilization of the provided DHT. No ML algorithm, regardless of complexity, can completely compensate for poor quality or largely missing data, though some imputation algorithms can remediate this gap to a degree. As such, active steps to ensure an adequate explanation of how to appropriately comply with a study protocol, perhaps with more planned remediation and outreach to study participants, are advisable.

Concerns around data privacy are always crucial in the world of DHT but may have added importance to individuals with PSD with a component of paranoid ideation. In addition to taking appropriate steps to ensure data privacy, additional steps of building trust with users and their community may help to assuage such fears.

Despite these limitations, there remains some optimism about the future of digital phenotyping and the application of ML to such data. Some current platforms, such as the mindLAMP digital platform, have demonstrated the importance of sharing back data in the interest of replicability. Moreover, it is important to acknowledge the ongoing work being conducted by the Accelerating Medicines Partnership Schizophrenia (AMP SCZ) program (Accelerating Medicines Partnership, n.d.). This substantial partnership spanning regulatory agencies in the United States and elsewhere around the world as well as several for profit and nonprofit organizations has been created with the aim of remediating the critical need for cost-effective treatments for schizophrenia. By collecting biomarker measures and digital assessments longitudinally, this collaboration aims to derive clinically useful screening algorithms and validate the efficacy of biomarkers in the CHR population. The ultimate aim is to improve pharmaceutical development in this high-risk population and may fill in the gaps posed by much of the existing research in this field through greater standardization.

As larger high-quality datasets become more readily available for the treatment of and screening for PSD, ML approaches can be tested against them. Over time, this may produce effective tools for screening for psychotic decompensation or diagnosing PSD. While the field of digital phenotyping, as it applies to PSD, is in its nascency, this is an exciting time for innovation and collaboration among those currently in the field through projects such as AMP SCZ. Given the substantial psychosocial, biological, and financial toll this condition can have on individuals and communities, it is imperative that we not exclude this particularly vulnerable population in such cutting-edge research.

The use of digital markers with ML provides an avenue to overcome many challenges faced in drug discovery and development, where conventional assessment of patients is restricted by limited time points and uncontrollable environmental impact (Baran et al., 2022). Using preclinical *in vivo* animal models, scalable digital monitoring technologies have shown promise in collecting translational digital biomarkers from subjects in a controlled environment, allowing for continuous monitoring and mitigating the effects of different environmental exposures on physiology and behavior, including those stemming from removal and handling (Baran et al., 2022). These novel approaches, combined with ML-based methods to enhance traditional preclinical studies, have the potential to bridge the gap between preclinical and clinical studies and generate viable avenues toward therapeutic development.

## Advancing drug discovery and treatment through machine learning and biomarkers in preclinical studies

Animal models serve as invaluable tools in neuropsychiatric research, providing controlled environments to explore disorder mechanisms and assess the efficacy and safety of potential treatments (Belzung and Lemoine, 2011; Chadman et al., 2009; Nestler and Hyman, 2010; Wendler and Wehling, 2010). These models play a pivotal role in elucidating biomarker causality, validating human-identified biomarkers, and discovering novel ones by replicating phenotypic aspects of human psychiatric disorders (Wendler and Wehling, 2010; Humer et al., 2020; Markou et al., 2009; Nestler and Hyman, 2010; Tomoda et al., 2016). Moreover, animal models facilitate the assessment of novel therapeutic agents based on biomarker profiles using techniques practically challenging in human studies (Humer et al., 2020).

ML can accelerate and refine the preclinical research process by analyzing complex datasets, enhancing biomarker identification and sensitivity, and improving their translational value. Leveraging the extensive data collection potential from preclinical models as input for ML, researchers can take a data-driven approach to target specific aspects of the PSD, identify novel biomarkers and evaluate treatment efficacy. Importantly, ML has emerged as a powerful tool in enhancing the precision and speed of preclinical behavioral research, where the complexity and amount of behavioral and neural data collected using novel techniques has advanced beyond the capabilities of traditional analytical methods (Valletta et al., 2017).

Semi-Supervised Learning Techniques: Semi-supervised learning methods offer a valuable approach for biomarker identification by effectively utilizing both labeled and unlabeled data, particularly beneficial when labeled data is limited or costly to obtain (Y.C.a et al., 2018). The integration of supervised and unsupervised methods in semi-supervised learning addresses the limitations of each, enhancing the potential for comprehensive biomarker discovery efforts (Whiteway et al., 2021). Traditional analyses of behavioral data require time-consuming and error-prone human video annotation, limiting the scale and complexity of behavioral datasets (Huk et al., 2018). The 3-Dimensional Aligned Neural Network for Computational Ethology (DANNCE) program employs semi-supervised learning for pose estimation in animal behavior videos, overcoming challenges of traditional methods and providing a more comprehensive representation of behavior by enabling the prediction of animal poses in videos with missing values due to object occlusion and removing the need for animals to wear invasive markers (Karashchuk et al., 2021; Dunn et al., 2021). This approach may aid in evaluating therapeutic agent efficacy in schizophrenia by evaluating alterations to movement-related biomarkers, such as locomotor hyperactivity in response to novel environments (Monfil et al., 2018).

Supervised Learning Techniques: Preclinical datasets are often characterized by high-dimensionality and nonlinear dependencies across multiple variables, posing significant challenges for traditional statistical approaches (Valletta et al., 2017). Supervised learning techniques, such as LASSO logistic regression, random forest, and SVM recursive feature elimination (SVM-RFE), provide a promising avenue for systematically selecting features with the highest predictive power for psychiatric disorder diagnosis or treatment outcomes in animal models (Dwiel et al., 2019; Orrù et al., 2012; Valletta et al., 2017). This approach has been illustrated in studies using feature selection methods to identify biomarkers from large datasets generated through high-throughput technologies such as proteomics, genetic profiling, transcriptomics and metabolomics (Stapleton et al., 2019; Zhang et al., 2023; Zhang et al., 2020). For example, Zhu et al. 2023, employed LASSO, random forest, and SVM-RFE on human gene expression data from schizophrenia patients to identify CLIC3 as a potential genetic biomarker (Zhu et al. 2023). Validation in an MK-801 rat model of schizophrenia revealed reduced CLIC3 levels in MK-801 rats, which could be reversed by risperidone treatment (Zhu et al. 2023). ML techniques also hold promise in identifying systems-level neural biomarkers, such as those capable of predicting appetitive behaviors in rodent models, thereby offering insights towards disorders like BPD and schizophrenia and the development of neuromodulation-based treatments (Dwiel et al., 2019; Horan et al., 2016).

Unsupervised Learning Techniques: Unsupervised learning techniques, including clustering algorithms and dimensionality reduction methods, provide a data-driven approach for discovering biomarkers in the absence of labeled outcome variables by identifying common features or grouping animals based on similarities in behavioral, neural, genetic, metabolic, or proteomic profiles (Valletta et al., 2017). Recent advances in multi-region recording techniques have facilitated the examination of brain-wide activity patterns influencing emotional behavior in animal studies (Mague et al., 2022).

Mague et al. (2022), applied discriminative cross-spectral factor analysis non-negative matrix factorization (dCSFA-NMF) to elucidate a neural network associated with individual variations in rodent social behavior, offering potential for identifying neural biomarkers in psychiatric disorders like schizophrenia, characterized by impaired social function, as seen in MAM-exposed rats showing altered social interactions, reduced interest in social stimuli, and changes in oxytocin and vasopressin levels (Mague et al., 2022; Potasiewicz et al., 2020). Additionally, a behaviorally focused approach called Motion Sequencing (MoSeq) combines 3D imaging and unsupervised ML to identify 3D mouse behavioral motifs and transitional patterns associated with genetic mutations or drug treatments in animal models of neuropsychiatric disorders (Wiltschko et al., 2020). By leveraging MoSeq to phenotype mice and assess the effects of pharmacological interventions, researchers can identify behavioral biomarkers to assess therapeutic agent efficacy and off-target effects, thereby advancing our pathophysiological understanding and informing potential treatments for neuropsychiatric disorders, including schizophrenia (Wiltschko et al., 2020).

Reinforcement Learning Techniques: Reinforcement learning (RL) algorithms, rooted in behavioral psychology principles, involve an agent interacting with an environment, receiving feedback as rewards or penalties, and iteratively adjusting its behavior to optimize outcomes (Morales and Escalante, 2022; Ribba et al., 2020). Following biomarker identification and validation through techniques such as supervised, unsupervised, and semi-supervised learning algorithms, iterative RL methods offer the potential for optimizing treatment dosing by dynamically adapting to individual responses based on biomarker profiles (Ribba et al., 2020). Liu et al. (2022), utilized RL to identify the minimal changes in microbe composition needed to shift predictions from obesity to health, suggesting its applicability in modulating microbial compositions based on biomarker profiles in disorders influenced by gut-brain interactions (Liu et al., 2022). Insights from Zheng et al. (2019), underscore the therapeutic potential of this framework, as germ-free mice receiving schizophrenia patient-derived microbiome transplants exhibited schizophrenia-like neurochemical and behavioral changes, suggesting microbiome-based interventions using RL could modulate neurological function (P. Zheng et al., 2019). Additionally, RL models have provided a foundational framework for understanding reward prediction errors (RPEs) in dopamine contexts, suggesting that the brain may represent future rewards not as a single mean but as a probability distribution, with diverse RPE representations observed across dopamine neurons in the mouse ventral tegmental area (Akam and Walton, 2021; Dabney et al., 2020). Schizophrenia’s underlying mechanism has been linked to RL deficits, including reduced learning rates, impaired value representation, aberrant salience attribution and reduced RPE processing, likely mediated by dopamine transmission as evidenced in rodent models (Boehme et al., 2015; Stopper and Floresco, 2015; Strauss et al., 2021).

The empirical study of RL disruptions using rodent models, coupled with RL algorithms and theories, provides a robust framework for understanding the neurobiological underpinnings of these disruptions in psychosis and schizophrenia, offering the potential for identifying biomarkers indicative of cognitive deficits with therapeutic implications.

Translating research findings from animal studies to human outcomes is crucial for successful therapeutic intervention development but requires careful validation and interpretation due to species differences and the artificial nature of disease models, often leading to limited predictive validity in clinical trials (Normand et al., 2018; Wendler and Wehling, 2010). ML methods offer a promising solution by integrating species-specific differences into data interpretation (Normand et al., 2018). The Found in Translation (FIT) method, proposed by Normand et al. 2018, leverages public gene expression data to extrapolate findings from mouse experiments to equivalent human conditions, enhancing the identification of disorder-associated genes (Normand et al., 2018). The mouse2man program, part of the FIT framework, utilizes human-mouse expression data to predict human disorder effect sizes, enhancing the translational potential of findings from animal studies and guiding the development of targeted therapies and interventions in humans (Normand et al., 2018). Similarly, Iwata et al. (2021), developed a multimodal learning method based on deep learning to predict total drug clearance in humans based on animal data (Iwata et al., 2021). By integrating chemical structure data and rat clearance rates, this method provides insights into the factors influencing drug clearance, aiding in the prediction of human drug metabolism pathways (Iwata et al., 2021). These innovative approaches highlight the potential of ML to bridge the gap between animal and human research, offering opportunities for the development of more targeted treatments and improved prediction accuracy of drug efficacy and toxicity (Iwata et al., 2021). Future research integrating ML techniques holds promise for advancing translational research in neuropsychiatric disorders, particularly in biomarker discovery and validation, ultimately enhancing the development of effective therapeutic interventions.

## Discussion

Numerous implications and applications arise from leveraging ML approaches in the psychosis spectrum. As we have shown, these approaches enable real-time integration of diverse input sources, ranging from cognitive to digital markers (Cascella et al., 2021; Krauss et al., 2021). We summarize key points in Box 1.

Overall, we have highlighted how ML excels in biomarker discovery in a myriad of ways that benefit psychosis research. In particular, we have shown the strengths of ML-based approaches in several specific domains, and the limitations of current approaches along with areas for improvement. With regard to cognition, ML has proven to be especially useful for pre-onset assessment. In particular, it has been effective in diagnosis, differentiating CHR from HC, and transitioning from CHR to frank psychosis. ML has also provided new avenues to simultaneously combine information from multiple cognitive domains and use their combined information to predict functional outcomes.

Many PSD-related cognitive domains show notable overlap with other domains while also providing unique information, and ML is particularly well suited to address such multicollinearity. neuroimaging-based features have demonstrated particular promise, especially using structural data, in tandem with ML. Specific applications that we highlight above include differentiation based on brain structure for both disorder classifications (e.g., schizophrenia versus BPD, and HC) as well as subtyping within PSD. This has high potential in forming the foundation for personalized medicine approaches where patients can be grouped based on neuroimaging features resulting in neuro-derived psychosis subtypes (Zhang et al. 2023). This approach would dovetail with the FC-based approaches to improve treatment prediction. We also discussed the importance of ML in identifying electrophysiological biomarkers that can be especially useful for identifying PSD’s perceptual aberrations. Additionally, we underscore findings from digital markers and animal-based models that are informative both for contemporary and potential future uses. Digital markers, for example, are being integrated with biobehavioral markers in a number of creative ways, including real-time monitoring of biomarkers that can provide feedback relevant to medicinal efficacy, as well as adherence measurement that is based on both medication use and multivariate biomarker data sources that are combined using ML. We also discussed innovative applications of accelerometer and GPS data that naturally conform to definitions of negative symptoms, and are particularly exciting in terms of continuous assessment of such symptoms and rapid identification of potential symptom progression. We also mentioned how ML can help accelerate the translational pipeline from animal research to human application (Brubaker and Lauffenburger, 2020). ML has been particularly useful in identifying relevant biomarkers that can inform unique translational pathways to humans.

We also note a number of high-level themes that cut across each of these biomarker domains. First, ML is effective at amalgamating a myriad of small effects, which, while individually modest, collectively yield significant clinical insights. The nature of some ML approaches, such as clustering, can circumvent the need for a priori hypotheses, though they are most effective when synergizing with hypothesis-driven approaches based on established clinical paradigms rather than supplanting them entirely. Second, such approaches can extend beyond individual patient surveillance. Ongoing efforts to mine electronic medical records for overarching trends and personalized patient applications are particularly relevant in connecting pre-onset symptoms to established psychosis, which can occur through the use of extensive historical data (Irving et al., 2021). Third, data-driven clustering approaches can also identify patient subgroups who differentially respond to treatment, exemplifying a precision medicine approach. In our review of MRI-based methods, we report on ongoing work that used structural MRI to achieve this end. Applying this approach to other biomarkers could yield additional benefits. Fourth, at the individual patient level, RL arises as a powerful instrument for dynamically fine-tuning clinical strategies over time, creating customized patient care trajectories (Spilka et al., 2023). This dynamic approach allows for the adaptation of treatment plans based on ongoing patient responses and changing symptomatology, leading to more effective and personalized care (Benoit et al., 2020b). Fifth, ML is effective at integrating advanced neuroimaging techniques, such as FC analysis and DTI, with clinical and cognitive assessment. This unified approach can offer unprecedented insights into the underlying neurobiology of psychosis. By elucidating the complex neural circuits implicated in the disorder, these techniques provide a foundation for the development of targeted interventions aimed at restoring aberrant brain function.

## Future directions

To maximize the potential of these exciting new approaches, several barriers need to be addressed. First, the reliability of defining various psychotic phenomena often remains inadequate. Based on the reliability observed in Diagnostic and Statistical Manual of Mental Disorders-5 field trials (Regier et al., 2013), the effect sizes of prediction models for multiple psychotic disorders are substantially attenuated (De Nadai et al., 2022), necessitating increased sample sizes for effect detection. This not only slows down new developments but also introduces confusion due to inconsistent detection of findings.

Moreover, there is a pressing need to significantly elevate standards for model performance to align with those established in other scientific domains. The aforementioned subgrouping methods, as well as mixture modeling along with latent variable models, can help increase precision in target definition. These approaches complement the National Institute of Mental Health Research Domain Criteria (RDoC), which deconstructs complex behaviors into specific endophenotypes and often use continuous rather than categorical target definitions (Morris et al., 2022). ML is well- equipped to handle multimodal RDoC data (Cuthbert, 2020), and it integrates effectively with traditional approaches.

It is important to note that while these approaches can address a large number of objective biobehavioral markers, PSD diagnoses inherently involve subjective elements. In clinical research applications, objective markers are ultimately linked to subjectively defined psychotic phenomena. Therefore, it is challenging to fully obviate the need for improvements in psychiatric nosology. Improving precision in this subjective domain can help reduce noise in data and increase the strength of the signal when connecting objective markers to subjectively defined psychotic phenomena.

At present, many ML approaches are considered “black box” methodologies, (Watson et al., 2019), this poses significant challenges, especially in clinical settings, where transparency and interpretability are paramount. Additionally, if there is model bias against a specific demographic group, identifying the source of bias within the model becomes difficult or even impossible, which raises ethical concerns regarding fairness and equity in mental healthcare. This lack of transparency is also problematic in clinical applications. If a model generates incorrect recommendations and interventions, it can lead to clinical harm, jeopardizing patient safety and well-being. In response to these challenges, a range of causal ML techniques are under active development (Richens et al., 2020; Wager and Athey, 2018). These methods aim to elucidate specific causal relationships between predictors and outcomes within complex datasets, providing a more interpretable framework for understanding and acting upon the insights derived from ML models. However, careful consideration of their suitability for clinical purposes is essential, as the adoption of new technologies must balance the need for transparency and interpretability with the demand for predictive accuracy and generalizability in real-world settings.

## Conclusion

Overall, our objective in this review has been to highlight the state-of-the-art use of ML in biomarker discovery, validation, and utility in the psychosis spectrum. We have also described current limitations and potential paths forward. ML-based approaches can complement current research and clinical practice, and they provide new opportunities that are not possible with conventional methods. In tandem with innovative multimodal biomarker data collection procedures, they provide great promise. While there remain key questions to be answered with regard to applied methodology and clinical application, we have highlighted ongoing efforts to address them that have demonstrated several initial gains. This nascent field has already produced many successes, and patients who experience psychotic symptoms stand to benefit greatly in the coming years.

## Box 1

**Table:** Biomarker discovery in the psychosis spectrum.

Modality	Summary

Cognition	Although cognition is typically considered alongside other predictors in the psychosis literature, key applications of cognition within ML frameworks in these populations include prediction of (1) diagnosis, (2) treatment response, (3) clinical outcomes, (4) functional outcomes, and (5) transition to frank psychosis. Several studies indicate that cognitive data have utility for the prediction of PSD versus HC, and addition of cognitive variables to existing models typically improves their performance. However, prediction of PSD versus non-psychotic disorders using cognitive measures is equivocal. Cognitive data have been used in models that successfully differentiate CHR from HC, and CHR individuals who transition to full-threshold psychosis from those who do not. Findings appear to favor the integration of neurocognitive data into larger clinical models in order to improve predictive ability, rather than unimodal cognitive models. Cognitive variables also contribute to the prediction of clinical and functional outcomes in CHR and FEP, although they are not sufficient on their own and often do not surpass clinical measures.
MRI	Neuroimaging research has yielded crucial insights into PSD pathophysiology, revealing structural and functional brain aberrations. ML techniques applied to MRI data show promise in distinguishing PSD from healthy controls, identifying subgroups, and predicting treatment outcomes. Classification accuracies generally range from 70 % to 85 %. Multimodal approaches tend to improve performance. Functional connectivity patterns, especially involving the striatum, show potential in predicting antipsychotic response. While promising, challenges remain in translating these approaches to clinical utility, including generalizability across sites and scanners.
EEG	From EEG and ERP, a number of biomarkers, including the ASSR, gamma band oscillations (30–80 Hz), MMN, sensory gating/P50, P300, and N100, are robust observations in PSD. Applying ML to these paradigms allows for a deeper understanding of the neurobiological mechanisms and heterogeneity underlying psychosis, facilitates early and accurate diagnosis, supports personalized treatment approaches, and improves the overall prediction of patients’ responses to treatment or prognosis. Both classical ML algorithms and deep learning algorithms like CNNs have been applied to EEG and ERP to this end. Future work exploring EEG and ERP at the whole scalp level holds the potential to pinpoint novel biomarkers for personalized treatment.
Digital Markers	Digital interventions for use in the screening and treatment of PSD include a wide range of existent technologies that take advantage of digital biomarkers. These technologies, which include software, physical sensors, and computing platforms, offer promising potential in monitoring PSD, yet require regulatory verification and usability evaluation. EMAs and wearable technologies have demonstrated their efficacy in tracking symptoms of schizophrenia, while digital phenotyping, combining active and passive data collection, shows promise in predicting relapses. However, ML algorithms, although promising for trend identification, face challenges due to small sample sizes and lack of standardization.Research has identified that digital phenotypic markers for PSD, such as frequency of text messaging and mobility are linked to symptom severity. Sleep quality and medication adherence are important factors, with medication non-adherence being a significant relapse predictor. Despite the potential of some of the tools we mentioned in this review, challenges in ethical considerations, data privacy, and participant engagement remain, especially for individuals with severe PSD symptoms.The digital phenotyping field, especially in PSD, is still in its early stages but holds great promise for innovation and collaboration. Overcoming current limitations can lead to meaningful advancements in PSD diagnosis and treatment, ultimately reducing the psychosocial, biological, and financial burdens of this condition on individuals and communities.
Preclinical Research	Despite advances in ML that enhance the identification and assessment of biomarker profiles in clinical practice, preclinical studies in controlled environments remain essential. ML enhances traditional preclinical research by improving the scale of behavioral data analysis, minimizing the “white coat effect” through non-invasive monitoring, and enabling more sensitive and accurate evaluations of pharmacological interventions and potential off-target effects. Additionally, ML aids in identifying PSD biomarkers from the high-throughput studies possible in preclinical research, developing novel treatment strategies, uncovering mechanisms contributing to PSD development, and ultimately improving the translatability of genetic and drug metabolism findings from preclinical studies to clinical applications.

## References

[R1] Accelerating Medicines Partnership. (n.d.). SCHIZOPHRENIA. Accelerating Medicines Partnership. https://www.ampscz.org/.

[R2] Afonso P, Brissos S, Cañas F, Bobes J, Bernardo-Fernandez I, 2014. Treatment adherence and quality of sleep in schizophrenia outpatients. Int. J. Psychiatry Clin. Pract. 18 (1), 70–76. 10.3109/13651501.2013.845219. 24047426

[R3] Ahmedt-Aristizabal D, Fernando T, Denman S, Robinson JE, Sridharan S, Johnston PJ, Laurens KR, Fookes C, 2021. Identification of children at risk of Schizophrenia via deep learning and EEG responses. Article 1 IEEE J. Biomed. Health Inform. 25 (1). 10.1109/JBHI.2020.2984238. 32310808

[R4] Akam T, Walton ME, 2021. What is dopamine doing in model-based reinforcement learning? Curr. Opin. Behav. Sci. 38, 74–82. 10.1016/j.cobeha.2020.10.010. 37082448 PMC7614453

[R5] Ambrosen KS, Skjerbæk MW, Foldager J, Axelsen MC, Bak N, Arvastson L, Christensen SR, Johansen LB, Raghava JM, Oranje B, Rostrup E, Nielsen MØ, Osler M, Fagerlund B, Pantelis C, Kinon BJ, Glenthøj BY, Hansen LK, Ebdrup BH, 2020. A machine-learning framework for robust and reliable prediction of short- and long-term treatment response in initially antipsychotic-naïve schizophrenia patients based on multimodal neuropsychiatric data. Transl. Psychiatry 10 (1), 276. 10.1038/s41398-020-00962-8. 32778656 PMC7417553

[R6] American Psychiatric Association, & American Psychiatric Association, 2013. Diagnostic and Statistical Manual of Mental Disorders: DSM-5, 5th ed. American Psychiatric Association.

[R7] Baran SW, Bratcher N, Dennis J, Gaburro S, Karlsson EM, Maguire S, Makidon P, Noldus LPJJ, Potier Y, Rosati G, Ruiter M, Schaevitz L, Sweeney P, LaFollette MR, 2022. Emerging role of translational digital biomarkers within home cage monitoring technologies in preclinical drug discovery and development. Front. Behav. Neurosci. 15. 10.3389/fnbeh.2021.758274. PMC8885444 35242017

[R8] Barch DM, Bustillo J, Gaebel W, Gur R, Heckers S, Malaspina D, Owen MJ, Schultz S, Tandon R, Tsuang M, Van Os J, Carpenter W, 2013. Logic and justification for dimensional assessment of symptoms and related clinical phenomena in psychosis: relevance to DSM-5. Schizophr. Res. 150 (1), 15–20. 10.1016/j.schres.2013.04.027. 23706415

[R9] Barros C, Silva CA, Pinheiro AP, 2021. Advanced EEG-based learning approaches to predict schizophrenia: Promises and pitfalls. Artif. Intell. Med. 114, 102039 10.1016/j.artmed.2021.102039. 33875158

[R10] Belzung C, Lemoine M, 2011. Criteria of validity for animal models of psychiatric disorders: Focus on anxiety disorders and depression. Biol. Mood Anxiety Disord. 1, 9. 10.1186/2045-5380-1-9. 22738250 PMC3384226

[R11] Benoit J, Onyeaka H, Keshavan M, Torous J, 2020a. Systematic review of digital phenotyping and machine learning in psychosis spectrum illnesses. Harv. Rev. Psychiatry 28 (5), 296–304. 10.1097/HRP.0000000000000268. 32796192

[R12] Benoit J, Onyeaka H, Keshavan M, Torous J, 2020b. Systematic review of digital phenotyping and machine learning in psychosis spectrum illnesses. Harv. Rev. Psychiatry 28 (5), 296–304. 10.1097/HRP.0000000000000268. 32796192

[R13] Boehme R, Deserno L, Gleich T, Katthagen T, Pankow A, Behr J, Buchert R, Roiser JP, Heinz A, Schlagenhauf F, 2015. Aberrant salience is related to reduced reinforcement learning signals and elevated dopamine synthesis capacity in healthy adults. J. Neurosci. 35 (28), 10103–10111. 10.1523/JNEUROSCI.0805-15.2015. 26180188 PMC6605337

[R14] Bolt LK, Amminger GP, Farhall J, McGorry PD, Nelson B, Markulev C, Yuen HP, Schäfer MR, Mossaheb N, Schlögelhofer M, Smesny S, Hickie IB, Berger GE, Chen EYH, De Haan L, Nieman DH, Nordentoft M, Riecher-Rössler A, Verma S, Allott KA, 2019. Neurocognition as a predictor of transition to psychotic disorder and functional outcomes in ultra-high risk participants: findings from the NEURAPRO randomized clinical trial. Schizophr. Res. 206, 67–74. 10.1016/j.schres.2018.12.013. 30558978

[R15] Bora E, Murray RM, 2014. Meta-analysis of cognitive deficits in ultra-high risk to psychosis and first-episode psychosis: do the cognitive deficits progress over, or after, the onset of psychosis. Schizophr. Bull. 40 (4), 744–755. 10.1093/schbul/sbt085. 23770934 PMC4059428

[R16] Bowie CR, Harvey PD, 2006. Cognitive deficits and functional outcome in schizophrenia. Neuropsychiatr. Dis. Treat. 2 (4), 531–536. 10.2147/nedt.2006.2.4.531. 19412501 PMC2671937

[R17] Brandizzi M, Valmaggia L, Byrne M, Jones C, Iwegbu N, Badger S, McGuire P, Fusar-Poli P, 2015. Predictors of functional outcome in individuals at high clinical risk for psychosis at six years follow-up. J. Psychiatr. Res. 65, 115–123. 10.1016/j.jpsychires.2015.03.005. 25837413

[R18] Brubaker DK, Lauffenburger DA, 2020. Translating preclinical models to humans. Science 367 (6479), 742–743. 10.1126/science.aay8086. 32054749

[R19] Buck B, Munson J, Chander A, Wang W, Brenner CJ, Campbell AT, Ben-Zeev D, 2022. The relationship between appraisals of auditory verbal hallucinations and real- time affect and social functioning. Schizophr. Res. 250, 112–119 10.1016/j.schres.2022.10.015. 36399900 PMC9750498

[R20] Buck B, Scherer E, Brian R, Wang R, Wang W, Campbell A, Choudhury T, Hauser M, Kane JM, Ben-Zeev D, 2019. Relationships between smartphone social behavior and relapse in schizophrenia: a preliminary report. Schizophr. Res. 208, 167–172. 10.1016/j.schres.2019.03.014. 30940400 PMC6580857

[R21] Cao H, Lencz T, Gallego JA, Rubio JM, John M, Barber AD, Birnbaum ML, Robinson DG, Malhotra AK, 2023. A functional connectome-based neural signature for individualized prediction of antipsychotic response in first-episode psychosis. Am. J. Psychiatry 180 (11), 827–835. 10.1176/appi.ajp.20220719. 37644811 PMC11104773

[R22] Carrión R, McLaughlin D, Auther A, Olsen R, Correll C, Cornblatt B, 2015. The impact of psychosis on the course of cognition: a prospective, nested case-control study in individuals at clinical high-risk for psychosis. Psychol. Med. 45 (15), 3341–3354. 26169626 10.1017/S0033291715001233 PMC4790441

[R23] Cascella N, Butala AA, Mills K, Kim MJ, Salimpour Y, Wojtasievicz T, Hwang B, Cullen B, Figee M, Moran L, Lenz F, Sawa A, Schretlen DJ, Anderson W, 2021. Deep brain stimulation of the substantia nigra pars reticulata for treatment-resistant schizophrenia: a case report. Biol. Psychiatry 90 (10), e57–e59. 10.1016/j.biopsych.2021.03.007. 33906736

[R24] Catalan A, Salazar de Pablo G, Aymerich C, Damiani S, Sordi V, Radua J, Oliver D, McGuire P, Giuliano AJ, Stone WS, Fusar-Poli P, 2021. Neurocognitive functioning in individuals at clinical high risk for psychosis: a systematic review and meta-analysis. JAMA Psychiatry 78 (8), 859–867. 10.1001/jamapsychiatry.2021.1290. 34132736 PMC8209603

[R25] Cella M, Okruszek Ł, Lawrence M, Zarlenga V, He Z, Wykes T, 2018. Using wearable technology to detect the autonomic signature of illness severity in schizophrenia. Schizophr. Res. 195, 537–542. 10.1016/j.schres.2017.09.028. 28986005

[R26] Chadman KK, Yang M, Crawley JN, 2009. Criteria for validating mouse models of psychiatric diseases. Am. J. Med. Genet. Part B Neuropsychiatr. Genet.: Off. Publ. Int. Soc. Psychiatr. Genet. 150B (1), 1–11. 10.1002/ajmg.b.30777. PMC2914616 18484083

[R27] Chand GB, Dwyer DB, Erus G, Sotiras A, Varol E, Srinivasan D, Doshi J, Pomponio R, Pigoni A, Dazzan P, Kahn RS, Schnack HG, Zanetti MV, Meisenzahl E, Busatto GF, Crespo-Facorro B, Pantelis C, Wood SJ, Zhuo C, Davatzikos C, 2020. Two distinct neuroanatomical subtypes of schizophrenia revealed using machine learning. Brain 143 (3), 1027–1038. 10.1093/brain/awaa025. 32103250 PMC7089665

[R28] Chang Q, Liu M, Tian Q, Wang H, Luo Y, Zhang J, Wang C, 2019. EEG-based brain functional connectivity in first-episode schizophrenia patients, ultra-high-risk individuals, and healthy controls during P50 suppression. Front. Hum. Neurosci. 13, 379. 10.3389/fnhum.2019.00379. 31803031 PMC6870009

[R29] Chen J, Patil KR, Weis S, Sim K, Nickl-Jockschat T, Zhou J, Aleman A, Sommer IE, Liemburg EJ, Hoffstaedter F, Habel U, Derntl B, Liu X, Fischer JM, Kogler L, Regenbogen C, Diwadkar VA, Stanley JA, Riedl V, Visser E, 2020. Neurobiological divergence of the positive and negative schizophrenia subtypes identified on a new factor structure of psychopathology using non-negative factorization: an international machine learning study. Biol. Psychiatry 87 (3), 282–293. 10.1016/j.biopsych.2019.08.031. 31748126 PMC6946875

[R30] Chopra S, Levi PT, Holmes A, Orchard ER, Segal A, Francey SM, O’Donoghue B, Cropley VL, Nelson B, Graham J, Baldwin L, Yuen HP, Allott K, Alvarez- Jimenez M, Harrigan S, Pantelis C, Wood SJ, McGorry P, Fornito A, 2023. Brain-wide disruptions of anatomical connectivity in antipsychotic-naïve first episode psychosis [Preprint]. Psychiatry Clin. Psychol. 10.1101/2023.11.10.23298391. 39069164

[R31] Clementz BA, Parker DA, Trotti RL, McDowell JE, Keedy SK, Keshavan MS, Pearlson GD, Gershon ES, Ivleva EI, Huang L-Y, Hill SK, Sweeney JA, Thomas O, Hudgens-Haney M, Gibbons RD, Tamminga CA, 2022. Psychosis biotypes: replication and validation from the B-SNIP consortium. Schizophr. Bull. 48 (1), 56–68. 10.1093/schbul/sbab090. 34409449 PMC8781330

[R32] Clementz BA, Sweeney JA, Hamm JP, Ivleva EI, Ethridge LE, Pearlson GD, Keshavan MS, Tamminga CA, 2016. Identification of distinct psychosis biotypes using brain-based biomarkers. Am. J. Psychiatry 173 (4), 373–384. 26651391 10.1176/appi.ajp.2015.14091200 PMC5314432

[R33] Cohen A, Naslund JA, Chang S, Nagendra S, Bhan A, Rozatkar A, Thirthalli J, Bondre A, Tugnawat D, Reddy PV, Dutt S, Choudhary S, Chand PK, Patel V, Keshavan M, Joshi D, Mehta UM, Torous J, 2023. Relapse prediction in schizophrenia with smartphone digital phenotyping during COVID-19: a prospective, three-site, two-country, longitudinal study. Schizophrenia 9 (1), 6. 10.1038/s41537-023-00332-5. 36707524 PMC9880926

[R34] Commissioner, O. of the (n.d.). Focus area: Digital health technologies. U. S. Food and Drug Administration. https://www.fda.gov/science-research/focus-areas-regulatory-science-report/focus-area-digital-health-technologies.

[R35] Cuthbert BN, 2020. The role of RDoC in future classification of mentaldisorders. Dialog −. Clin. Neurosci. 22 (1), 81–85. 10.31887/DCNS.2020.22.1/bcuthbert. PMC7365298 32699508

[R36] Dabney W, Kurth-Nelson Z, Uchida N, Starkweather CK, Hassabis D, Munos R, Botvinick M, 2020. A distributional code for value in dopamine-based reinforcement learning. Article 7792 Nature 577 (7792). 10.1038/s41586-019-1924-6. PMC7476215 31942076

[R37] De Nadai AS, Hu Y, Thompson WK, 2022. Data pollution in neuropsychiatry—an under-recognized but critical barrier to research progress. JAMA Psychiatry 79 (2), 97. 10.1001/jamapsychiatry.2021.2812. 34851392

[R38] de Nijs J, Burger TJ, Janssen RJ, Kia SM, van Opstal DPJ, de Koning MB, de Haan L, Cahn W, Schnack HG, 2021. Individualized prediction of three- and six-year outcomes of psychosis in a longitudinal multicenter study: a machine learning approach. Npj Schizophr. 7 (1). Article 1. 10.1038/s41537-021-00162-3. 34215752 PMC8253813

[R39] Del Fabro L, Bondi E, Serio F, Maggioni E, D’Agostino A, Brambilla P, 2023. Machine learning methods to predict outcomes of pharmacological treatment in psychosis. Article 1 Transl. Psychiatry 13 (1). 10.1038/s41398-023-02371-z. 36864017 PMC9981732

[R40] Depp CA, Bashem J, Moore RC, Holden JL, Mikhael T, Swendsen J, Harvey PD, Granholm EL, 2019. GPS mobility as a digital biomarker of negative symptoms in schizophrenia: a case control study. Npj Digit. Med. 2 (1), 108 10.1038/s41746-019-0182-1. 31728415 PMC6841669

[R41] Di Capite S, Upthegrove R, Mallikarjun P, 2018. The relapse rate and predictors of relapse in patients with first-episode psychosis following discontinuation of antipsychotic medication. Early Interv. Psychiatry 12 (5), 893–899. 10.1111/eip.12385. 27734591

[R42] Doan NT, Kaufmann T, Bettella F, Jørgensen KN, Brandt CL, Moberget T, Alnæs D, Douaud G, Duff E, Djurovic S, Melle I, Ueland T, Agartz I, Andreassen OA, Westlye LT, 2017. Distinct multivariate brain morphological patterns and their added predictive value with cognitive and polygenic risk scores in mental disorders. NeuroImage: Clin. 15, 719–731. 10.1016/j.nicl.2017.06.014. 28702349 PMC5491456

[R43] Donchin E, Ritter W, McCallum C, 1978. Cognitive psychophysiology: the endogenous components of the ERP. Event-Relat. Brain Potentials Man 349–441. 10.1016/B978-0-12-155150-6.X5001-9.

[R44] Du Y, Hao H, Wang S, Pearlson GD, Calhoun VD, 2020. Identifying commonality and specificity across psychosis sub-groups via classification based on features from dynamic connectivity analysis. NeuroImage: Clin. 27, 102284 10.1016/j.nicl.2020.102284. 32563920 PMC7306624

[R45] Du Y, He X, Kochunov P, Pearlson G, Hong LE, Van Erp TGM, Belger A, Calhoun VD, 2022. A new multimodality fusion classification approach to explore the uniqueness of schizophrenia and autism spectrum disorder. Hum. Brain Mapp. 43 (12), 3887–3903. 10.1002/hbm.25890. 35484969 PMC9294304

[R46] Dunn TW, Marshall JD, Severson KS, Aldarondo DE, Hildebrand DGC, Chettih SN, Wang WL, Gellis AJ, Carlson DE, Aronov D, Freiwald WA, Wang F, Ölveczky BP, 2021. Geometric deep learning enables 3D kinematic profiling across species and environments. Article 5 Nat. Methods 18 (5). 10.1038/s41592-021-01106-6. 33875887 PMC8530226

[R47] Dwiel LL, Khokhar JY, Connerney MA, Green AI, Doucette WT, 2019. Finding the balance between model complexity and performance: Using ventral striatal oscillations to classify feeding behavior in rats. PLOS Comput. Biol. 15 (4), e1006838 10.1371/journal.pcbi.1006838. 31009448 PMC6497302

[R48] Dwyer DB, Cabral C, Kambeitz-Ilankovic L, Sanfelici R, Kambeitz J, Calhoun V, Falkai P, Pantelis C, Meisenzahl E, Koutsouleris N, 2018. Brain subtyping enhances the neuroanatomical discrimination of Schizophrenia. Schizophr. Bull. 44 (5), 1060–1069. 10.1093/schbul/sby008. 29529270 PMC6101481

[R49] Fernandes BS, Karmakar C, Tamouza R, Tran T, Yearwood J, Hamdani N, Laouamri H, Richard J-R, Yolken R, Berk M, Venkatesh S, Leboyer M, 2020. Precision psychiatry with immunological and cognitive biomarkers: a multi-domain prediction for the diagnosis of bipolar disorder or schizophrenia using machine learning. Transl. Psychiatry 10 (1), 162. 10.1038/s41398-020-0836-4. 32448868 PMC7246255

[R50] Fond G, Bulzacka E, Boucekine M, Schürhoff F, Berna F, Godin O, Aouizerate B, Capdevielle D, Chereau I, D’Amato T, Dubertret C, Dubreucq J, Faget C, Leignier S, Lançon C, Mallet J, Misdrahi D, Passerieux C, Rey R, Llorca PM, 2019. Machine learning for predicting psychotic relapse at 2 years in schizophrenia in the national FACE-SZ cohort. Prog. Neuro-Psychopharmacol. Biol. Psychiatry 92, 8–18. 10.1016/j.pnpbp.2018.12.005. 30552914

[R51] Freedman R, 2014. А7-nicotinic acetylcholine receptor agonists for cognitive enhancement in schizophrenia. Annu. Rev. Med. 65, 245–261. 10.1146/annurev-med-092112-142937. 24111888

[R52] Fujiwara H, Yassin W, Murai T, 2015. Neuroimaging studies of social cognition in schizophrenia. Psychiatry Clin. Neurosci. 69 (5), 259–267. 10.1111/pcn.12258. 25418865

[R53] Gao J, Jiang R, Tang X, Chen J, Yu M, Zhou C, Wang X, Zhang H, Huang C, Yang Y, Zhang X, Cui Z, Zhang X, 2023. A neuromarker for deficit syndrome in schizophrenia from a combination of structural and functional magnetic resonance imaging. CNS Neurosci. Ther. 29 (12), 3774–3785. 10.1111/cns.14297. 37288482 PMC10651988

[R54] Ge R, Yu Y, Qi YX, Fan Y, Chen S, Gao C, Haas SS, New F, Boomsma DI, Brodaty H, Brouwer RM, Buckner R, Caseras X, Crivello F, Crone EA, Erk S, Fisher SE, Franke B, Glahn DC, Yu K, 2024. Normative modelling of brain morphometry across the lifespan with CentileBrain: algorithm benchmarking and model optimisation. Lancet Digit. Health 6 (3), e211–e221 10.1016/S2589-7500(23)00250-9. 38395541 PMC10929064

[R55] Glenthøj LB, Kristensen TD, Wenneberg C, Hjorthøj C, Nordentoft M, 2020. Investigating cognitive and clinical predictors of real-life functioning, functional capacity, and quality of life in individuals at ultra-high risk for psychosis. sgaa027 Schizophr. Bull. Open 1 (1). 10.1093/schizbullopen/sgaa027.

[R56] Gómez-gastiasoro A, Peña J, Zubiaurre-elorza L, Del Pino R, Ibarretxe-bilbao N, Ojeda N, 2020. Cognitive scores as a potential diagnostic tool in schizophrenia: the use of raw and discrepancy scores. Clin. Psychol. 24 (1), 73–81. 10.1111/cp.12191.

[R57] Gong W, Bai S, Zheng Y-Q, Smith SM, Beckmann CF, 2023. Supervised phenotype discovery from multimodal brain imaging. IEEE Trans. Med. Imaging 42 (3), 834–849. 10.1109/TMI.2022.3218720. 36318559 PMC7618907

[R58] Groves AR, Beckmann CF, Smith SM, Woolrich MW, 2011. Linked independent component analysis for multimodal data fusion. NeuroImage 54 (3), 2198–2217. 10.1016/j.neuroimage.2010.09.073. 20932919

[R59] Haining K, Brunner G, Gajwani R, Gross J, Gumley AI, Lawrie SM, Schwannauer M, Schultze-Lutter F, Uhlhaas PJ, 2021. The relationship between cognitive deficits and impaired short-term functional outcome in clinical high-risk for psychosis participants: a machine learning and modelling approach. Schizophr. Res. 231, 24–31. 10.1016/j.schres.2021.02.019. 33744682

[R60] Haining K, Gajwani R, Gross J, Gumley AI, Ince RAA, Lawrie SM, Schultze-Lutter F, Schwannauer M, Uhlhaas PJ, 2022. Characterising cognitive heterogeneity in individuals at clinical high-risk for psychosis: a cluster analysis with clinical and functional outcome prediction. Eur. Arch. Psychiatry Clin. Neurosci. 272 (3), 437–448. 10.1007/s00406-021-01315-2. 34401957 PMC8938352

[R61] Hedges EP, Dickson H, Tognin S, Modinos G, Antoniades M, van der Gaag M, de Haan L, McGorry P, Pantelis C, Riecher-Rossler A, Bressan R, Barrantes-Vidal N, Krebs M-O, Nordentoft M, Ruhrmann S, Sachs G, Rutten BP, van Os J, Valmaggia LR, Kempton MJ, 2022. Verbal memory performance predicts remission and functional outcome in people at clinical high-risk for psychosis. Schizophr. Res.: Cogn. 28, 100222 10.1016/j.scog.2021.100222. 35242602 PMC8861401

[R62] Henson P, Barnett I, Keshavan M, Torous J, 2020a. Towards clinically actionable digital phenotyping targets in schizophrenia. NPJ Schizophr. 6 (1), 13. 10.1038/s41537-020-0100-1. 32372059 PMC7200667

[R63] Henson P, Barnett I, Keshavan M, Torous J, 2020b. Towards clinically actionable digital phenotyping targets in schizophrenia. NPJ Schizophr. 6 (1), 13. 10.1038/s41537-020-0100-1. 32372059 PMC7200667

[R64] Honnorat N, Dong A, Meisenzahl-Lechner E, Koutsouleris N, Davatzikos C, 2019. Neuroanatomical heterogeneity of schizophrenia revealed by semi-supervised machine learning methods. Schizophr. Res. 214, 43–50. 10.1016/j.schres.2017.12.008. 29274735 PMC6013334

[R65] Horan WP, Wynn JK, Hajcak G, Altshuler L, Green MF, 2016. Distinct patterns of dysfunctional appetitive and aversive motivation in bipolar disorder versus schizophrenia: an event related potential study. J. Abnorm. Psychol. 125 (4), 576–587. 10.1037/abn0000142. 26845261 PMC4850103

[R66] Howes OD, Cummings C, Chapman GE, Shatalina E, 2023. Neuroimaging in schizophrenia: an overview of findings and their implications for synaptic changes. Neuropsychopharmacology 48 (1), 151–167 10.1038/s41386-022-01426-x. 36056106 PMC9700830

[R67] Huk A, Bonnen K, He BJ, 2018. Beyond trial-based paradigms: continuous behavior, ongoing neural activity, and natural stimuli. J. Neurosci. 38 (35), 7551–7558. 10.1523/JNEUROSCI.1920-17.2018. 30037835 PMC6113904

[R68] Humer E, Probst T, Pieh C, 2020. Metabolomics in psychiatric disorders: what we learn from animal models. Article 2 Metabolites 10 (2). 10.3390/metabo10020072. PMC7074444 32079262

[R69] Irving J, Patel R, Oliver D, Colling C, Pritchard M, Broadbent M, Baldwin H, Stahl D, Stewart R, Fusar-Poli P, 2021. Using natural language processing on electronic health records to enhance detection and prediction of psychosis risk. Schizophr. Bull. 47 (2), 405–414. 10.1093/schbul/sbaa126. 33025017 PMC7965059

[R70] Iwata H, Matsuo T, Mamada H, Motomura T, Matsushita M, Fujiwara T, Kazuya M, Handa K, 2021. Prediction of total drug clearance in humans using animal data: proposal of a multimodal learning method based on deep learning. J. Pharm. Sci. 110 (4), 1834–1841. 10.1016/j.xphs.2021.01.020. 33497658

[R71] Javitt DC, Freedman R, 2015. Sensory processing dysfunction in the personal experience and neuronal machinery of schizophrenia. Am. J. Psychiatry 172 (1), 17–31. 10.1176/appi.ajp.2014.13121691. 25553496 PMC4501403

[R72] Javitt D, Spencer Thaker, Winterer Hajos, 2008. Neurophysiological biomarkers for ´ drug development in schizophrenia. Article 1 Nat. Rev. Drug Discov. 7 (1). 10.1038/nrd2463. 18064038 PMC2753449

[R73] Jiang Y, Luo C, Wang J, Palaniyappan L, Chang X, Xiang S, Zhang J, Duan M, Huang H, Gaser C, Nemoto K, Miura K, Hashimoto R, Westlye LT, Richard G, Fernandez-Cabello S, Parker N, Andreassen OA, Kircher T, Feng J, 2023. Two neurostructural subtypes: Results of machine learning on brain images from 4,291 individuals with schizophrenia [Preprint]. Psychiatry Clin. Psychol. 10.1101/2023.10.11.23296862.

[R74] Jiang Y, Wang J, Zhou E, Palaniyappan L, Luo C, Ji G, Yang J, Wang Y, Zhang Y, Huang C-C, Tsai S-J, Chang X, Xie C, Zhang W, Lv J, Chen D, Shen C, Wu X, Zhang B, 2023. Neuroimaging biomarkers define neurophysiological subtypes with distinct trajectories in schizophrenia. … the ZIB Consortium Nat. Ment. Health 1 (3), 186–199. 10.1038/s44220-023-00024-0.

[R75] Kadakia A, Catillon M, Fan Q, Williams GR, Marden JR, Anderson A, Kirson N, Dembek C, 2022. The economic burden of schizophrenia in the United States. J. Clin. Psychiatry 83 (6). 10.4088/JCP.22m14458. 36244006

[R76] Karashchuk P, Tuthill JC, Brunton BW, 2021. The DANNCE of the rats: a new toolkit for 3D tracking of animal behavior. Article 5 Nat. Methods 18 (5). 10.1038/s41592-021-01110-w. 33875886

[R77] Keefe RSE, Fox KH, Harvey PD, Cucchiaro J, Siu C, Loebel A, 2011. Characteristics of the MATRICS Consensus Cognitive Battery in a 29-site antipsychotic schizophrenia clinical trial. Schizophr. Res. 125 (2–3), 161–168. 10.1016/j.schres.2010.09.015. 21075600

[R78] Keshavan MS, Collin G, Guimond S, Kelly S, Prasad KM, Lizano P, 2020. Neuroimaging in schizophrenia. Neuroimaging Clin. North Am. 30 (1), 73–83. 10.1016/j.nic.2019.09.007. PMC7724147 31759574

[R79] Kessler RC, Birnbaum H, Demler O, Falloon IRH, Gagnon E, Guyer M, Howes MJ, Kendler KS, Shi L, Walters E, Wu EQ, 2005. The prevalence and correlates of nonaffective psychosis in the national comorbidity survey replication (NCS-R). Biol. Psychiatry 58 (8), 668–676. 10.1016/j.biopsych.2005.04.034. 16023620 PMC2847859

[R80] Kirkpatrick B, Galderisi S, 2008. Deficit schizophrenia: an update. World Psychiatry 7 (3), 143–147. 10.1002/j.2051-5545.2008.tb00181.x. 18836581 PMC2559917

[R81] Koutsouleris N, Davatzikos C, Bottlender R, Patschurek-Kliche K, Scheuerecker J, Decker P, Gaser C, Möller H-J, Meisenzahl EM, 2012. Early recognition and disease prediction in the at-risk mental states for psychosis using neurocognitive pattern classification. Schizophr. Bull. 38 (6), 1200–1215. 10.1093/schbul/sbr037. 21576280 PMC3494049

[R82] Krauss JK, Lipsman N, Aziz T, Boutet A, Brown P, Chang JW, Davidson B, Grill WM, Hariz MI, Horn A, Schulder M, Mammis A, Tass PA, Volkmann J, Lozano AM, 2021. Technology of deep brain stimulation: Current status and future directions. Nat. Rev. Neurol. 17 (2), 75–87 10.1038/s41582-020-00426-z. 33244188 PMC7116699

[R83] Lakhtakia T, Bondre A, Chand PK, Chaturvedi N, Choudhary S, Currey D, Dutt S, Khan A, Kumar M, Gupta S, Nagendra S, Reddy PV, Rozatkar A, Scheuer L, Sen Y, Shrivastava R, Singh R, Thirthalli J, Tugnawat DK, Torous J, 2022. Smartphone digital phenotyping, surveys, and cognitive assessments for global mental health: Initial data and clinical correlations from an international first episode psychosis study. DIGITAL HEALTH 8, 205520762211337. 10.1177/20552076221133758. PMC9647298 36386246

[R84] Lalousis PA, Schmaal L, Wood SJ, Reniers RLEP, Barnes NM, Chisholm K, Griffiths SL, Stainton A, Wen J, Hwang G, Davatzikos C, Wenzel J, Kambeitz-Ilankovic L, Andreou C, Bonivento C, Dannlowski U, Ferro A, Lichtenstein T, Riecher-Rössler A, Upthegrove R, 2022. Neurobiologically based stratification of recent-onset depression and psychosis: identification of two distinct transdiagnostic phenotypes. Biol. Psychiatry 92 (7), 552–562. 10.1016/j.biopsych.2022.03.021. 35717212 PMC10128104

[R85] Lalousis PA, Wood SJ, Schmaal L, Chisholm K, Griffiths SL, Reniers RLEP, Bertolino A, Borgwardt S, Brambilla P, Kambeitz J, Lencer R, Pantelis C, Ruhrmann S, Salokangas RKR, Schultze-Lutter F, Bonivento C, Dwyer D, Ferro A, Haidl T, 2021. Heterogeneity and classification of recent onset psychosis and depression: a multimodal machine learning approach. … PRONIA Consortium Schizophr. Bull. 47 (4), 1130–1140. 10.1093/schbul/sbaa185. 33543752 PMC8266654

[R86] Lee HS, Kim JS, 2022. Implication of electrophysiological biomarkers in psychosis: focusing on diagnosis and treatment response. J. Pers. Med. 12 (1), 31. 10.3390/jpm12010031. 35055346 PMC8779239

[R87] Li A, Zalesky A, Yue W, Howes O, Yan H, Liu Y, Fan L, Whitaker KJ, Xu K, Rao G, Li J, Liu S, Wang M, Sun Y, Song M, Li P, Chen J, Chen Y, Wang H, Liu B, 2020. A neuroimaging biomarker for striatal dysfunction in schizophrenia. Nat. Med. 26 (4), 558–565. 10.1038/s41591-020-0793-8. 32251404

[R88] Liang S, Wang Q, Greenshaw AJ, Li X, Deng W, Ren H, Zhang C, Yu H, Wei W, Zhang Y, Li M, Zhao L, Du X, Meng Y, Ma X, Yan C-G, Li T, 2021. Aberrant triple-network connectivity patterns discriminate biotypes of first-episode medication-naive schizophrenia in two large independent cohorts. Neuropsychopharmacology 46 (8), 1502–1509 10.1038/s41386-020-00926-y. 33408329 PMC8208970

[R89] Lin A, Wood SJ, Nelson B, Brewer WJ, Spiliotacopoulos D, Bruxner A, Broussard C, Pantelis C, Yung AR, 2011. Neurocognitive predictors of functional outcome two to 13years after identification as ultra-high risk for psychosis. Schizophr. Res. 132 (1), 1–7. 10.1016/j.schres.2011.06.014. 21763109

[R90] Lin E, Lin C-H, Lane H-Y, 2021. Applying a bagging ensemble machine learning approach to predict functional outcome of schizophrenia with clinical symptoms and cognitive functions. Sci. Rep. 11 (1), 6922 10.1038/s41598-021-86382-0. 33767310 PMC7994315

[R91] Lindgren M, Holm M, Kieseppä T, Suvisaari J, 2020. Neurocognition and social cognition predicting 1-year outcomes in first-episode psychosis. Front. Psychiatry 11, 603933. 10.3389/fpsyt.2020.603933. 33343430 PMC7746550

[R92] Liu Y, Zhu J, Wang H, Lu W, LEE YK, Zhao J, Zhang H, 2022. Machine learning framework for gut microbiome biomarkers discovery and modulation analysis in large- scale obese population. BMC Genom. 23(1), 850 022–09087–2. 10.1186/s12864-. PMC9789565 36564713

[R93] Luo Y, Zhang J, Wang C, Zhao X, Chang Q, Wang H, Wang C, 2019. Discriminating schizophrenia disease progression using a P50 sensory gating task with dense-array EEG, clinical assessments, and cognitive tests. Article 5 Expert Rev. Neurother. 19 (5). 10.1080/14737175.2019.1601558. 31111773

[R94] Mague SD, Talbot A, Blount C, Walder-Christensen KK, Duffney LJ, Adamson E, Bey AL, Ndubuizu N, Thomas GE, Hughes DN, Grossman Y, Hultman R, Sinha S, Fink AM, Gallagher NM, Fisher RL, Jiang Y-H, Carlson DE, Dzirasa K, 2022. Brain-wide electrical dynamics encode individual appetitive social behavior. e7 Neuron 110 (10), 1728–1741. 10.1016/j.neuron.2022.02.016. 35294900 PMC9126093

[R95] Markou A, Chiamulera C, Geyer MA, Tricklebank M, Steckler T, 2009. Removing obstacles in neuroscience drug discovery: the future path for animal models. Neuropsychopharmacol.: Off. Publ. Am. Coll. Neuropsychopharmacol. 34 (1), 74–89. 10.1038/npp.2008.173. PMC2651739 18830240

[R96] McCutcheon RA, Keefe RSE, McGuire PK, 2023. Cognitive impairment in schizophrenia: Aetiology, pathophysiology, and treatment. Mol. Psychiatry 28 (5), 1902–1918. 10.1038/s41380-023-01949-9. 36690793 PMC10575791

[R97] Mehta UM, Ibrahim FA, Sharma MS, Venkatasubramanian G, Thirthalli J, Bharath RD, Bolo NR, Gangadhar BN, Keshavan MS, 2021. Resting-state functional connectivity predictors of treatment response in schizophrenia – a systematic review and meta-analysis. Schizophr. Res. 237, 153–165. 10.1016/j.schres.2021.09.004. 34534947

[R98] Meyer N, Joyce DW, Karr C, De Vos M, Dijk D-J, Jacobson NC, MacCabe JH, 2022. The temporal dynamics of sleep disturbance and psychopathology in psychosis: a digital sampling study. Psychol. Med. 52 (13), 2741–2750. 10.1017/S0033291720004857. 33431090 PMC9647520

[R99] Monfil T, Vázquez Roque RA, Camacho-Abrego I, Tendilla-Beltran H, Iannitti T, Meneses-Morales I, Aguilar-Alonso P, Flores G, Morales-Medina JC, 2018. Hyper-response to novelty increases c-fos expression in the hippocampus and prefrontal cortex in a rat model of schizophrenia. Neurochem. Res. 43 (2), 441–448. 10.1007/s11064-017-2439-x. 29214513

[R100] Morales EF, Escalante HJ, 2022. Chapter 6—A brief introduction to supervised, unsupervised, and reinforcement learning. In: Torres-García AA, Reyes-García CA, Villaseñor-Pineda L, Mendoza-Montoya O (Eds.), Biosignal Processing and Classification Using Computational Learning and Intelligence. Academic Press, pp. 111–129. 10.1016/B978-0-12-820125-1.00017-8.

[R101] Morris SE, Sanislow CA, Pacheco J, Vaidyanathan U, Gordon JA, Cuthbert BN, 2022. Revisiting the seven pillars of RDoC. BMC Med. 20 (1), 220. 10.1186/s12916-022-02414-0. 35768815 PMC9245309

[R102] Näätänen R, Todd J, Schall U, 2016. Mismatch negativity (MMN) as biomarker predicting psychosis in clinically at-risk individuals. Biol. Psychol. 116, 36–40. 10.1016/j.biopsycho.2015.10.010. 26542526

[R103] Nestler EJ, Hyman SE, 2010. Animal models of neuropsychiatric disorders. Nat. Neurosci, 13(10), Artic. 10. 10.1038/nn.2647. PMC3750731 20877280

[R104] Newson JJ, Thiagarajan TC, 2019. EEG frequency bands in psychiatric disorders: a review of resting state studies. Front. Hum. Neurosci. 12, 521. 10.3389/fnhum.2018.00521. 30687041 PMC6333694

[R105] Normand R, Du W, Briller M, Gaujoux R, Starosvetsky E, Ziv-Kenet A, Shalev-Malul G, Tibshirani RJ, Shen-Orr SS, 2018. Found In translation: a machine learning model for mouse-to-human inference. Article 12 Nat. Methods 15 (12). 10.1038/s41592-018-0214-9. PMC12396630 30478323

[R106] Oh H, Koyanagi A, Kelleher I, DeVylder J, 2018. Psychotic experiences and disability: findings from the collaborative psychiatric epidemiology surveys. Schizophr. Res. 193, 343–347. 10.1016/j.schres.2017.07.049. 28797526 PMC5912340

[R107] Olbrich S, Arns M, 2013. EEG biomarkers in major depressive disorder: discriminative power and prediction of treatment response. Int. Rev. Psychiatry (Abingdon, Engl.) 25 (5). Article 5. 10.3109/09540261.2013.816269. 24151805

[R108] Onitsuka T, Tsuchimoto R, Oribe N, Spencer KM, Hirano Y, 2022. Neuronal imbalance of excitation and inhibition in schizophrenia: a scoping review of gamma-band ASSR findings. Article 12 Psychiatry Clin. Neurosci. 76 (12). 10.1111/pcn.13472. 36069299

[R109] Orrù G, Pettersson-Yeo W, Marquand AF, Sartori G, Mechelli A, 2012. Using support vector machine to identify imaging biomarkers of neurological and psychiatric disease: a critical review. Neurosci. Biobehav. Rev. 36 (4), 1140–1152. 10.1016/j.neubiorev.2012.01.004. 22305994

[R110] Otsuka America Pharmaceutical, Inc (n.d.). AbilifyMyCite https://www.abilifymycite.com/.

[R111] Palaniyappan L, Deshpande G, Lanka P, Rangaprakash D, Iwabuchi S, Francis S, Liddle PF, 2019. Effective connectivity within a triple network brain system discriminates schizophrenia spectrum disorders from psychotic bipolar disorder at the single-subject level. Schizophr. Res. 214, 24–33. 10.1016/j.schres.2018.01.006. 29398207

[R112] Pan Y, Pu W, Chen X, Huang X, Cai Y, Tao H, Xue Z, Mackinley M, Limongi R, Liu Z, Palaniyappan L, 2020. Morphological profiling of schizophrenia: cluster analysis of mri-based cortical thickness data. Schizophr. Bull. 46 (3), 623–632. 10.1093/schbul/sbz112. 31901940 PMC7147597

[R113] Peritogiannis V, Ninou A, Samakouri M, 2022. Mortality in schizophrenia-spectrum disorders: recent advances in understanding and management. Healthcare 10 (12), 2366. 10.3390/healthcare10122366. 36553890 PMC9777663

[R114] Perrottelli A, Giordano GM, Brando F, Giuliani L, Mucci A, 2021. EEG-Based measures in at-risk mental state and early stages of schizophrenia: a systematic review. Front. Psychiatry 12, 653642. 10.3389/fpsyt.2021.653642. 34017273 PMC8129021

[R115] Pigoni A, Dwyer D, Squarcina L, Borgwardt S, Crespo-Facorro B, Dazzan P, Smesny S, Spaniel F, Spalletta G, Sanfelici R, Antonucci LA, Reuf A, Oeztuerk Oe.F., Schmidt A, Ciufolini S, Schönborn-Harrisberger F, Langbein K, Gussew A, Reichenbach JR, Brambilla P, 2021. Classification of first-episode psychosis using cortical thickness: a large multicenter MRI study. Eur. Neuropsychopharmacol. 47, 34–47. 10.1016/j.euroneuro.2021.04.002. 33957410

[R116] Pina-Camacho L, Garcia-Prieto J, Parellada M, Castro-Fornieles J, Gonzalez-Pinto AM, Bombin I, Graell M, Paya B, Rapado-Castro M, Janssen J, Baeza I, Pozo FD, Desco M, Arango C, 2015. Predictors of schizophrenia spectrum disorders in early-onset first episodes of psychosis: a support vector machine model. Eur. Child Adolesc. Psychiatry 24 (4), 427–440. 10.1007/s00787-014-0593-0. 25109600

[R117] Planchuelo-Gómez Á, Lubeiro A, Núñez-Novo P, Gomez-Pilar J, De Luis-García R, Del Valle P, Martín-Santiago Ó, Pérez-Escudero A, Molina V, 2020. Identificacion of MRI-based psychosis subtypes: Replication and refinement. Prog. Neuro- Psychopharmacol. Biol. Psychiatry 100, 109907. 10.1016/j.pnpbp.2020.109907. 32113850

[R118] Porter A, Fei S, Damme KSF, Nusslock R, Gratton C, Mittal VA, 2023. A meta-analysis and systematic review of single vs. Multimodal neuroimaging techniques in the classification of psychosis. Mol. Psychiatry 28 (8), 3278–3292. 10.1038/s41380-023-02195-9. 37563277 PMC10618094

[R119] Potasiewicz A, Holuj M, Litwa E, Gzielo K, Socha L, Popik P, Nikiforuk A, 2020. Social dysfunction in the neurodevelopmental model of schizophrenia in male and female rats: Behavioural and biochemical studies. Neuropharmacology 170, 108040. 10.1016/j.neuropharm.2020.108040. 32165218

[R120] Potter D, Summerfelt A, Gold J, Buchanan RW, 2005. Review of clinical correlates of p50 sensory gating abnormalities in patients with schizophrenia. Schizophr. Bull. 32 (4), 692–700. 10.1093/schbul/sbj050. PMC2632276 16469942

[R121] Price GD, Heinz MV, Zhao D, Nemesure M, Ruan F, Jacobson NC, 2022. An unsupervised machine learning approach using passive movement data to understand depression and schizophrenia. J. Affect. Disord. 316, 132–139. 10.1016/j.jad.2022.08.013. 35964770 PMC10064481

[R122] Ramyead A, Studerus E, Kometer M, Uttinger M, Gschwandtner U, Fuhr P, Riecher- Rössler A, 2016. Prediction of psychosis using neural oscillations and machine learning in neuroleptic-naïve at-risk patients. World J. Biol. Psychiatry, 17 (4), Artic. 4. 10.3109/15622975.2015.1083614. 26453061

[R123] Ranlund S, Nottage J, Shaikh M, Dutt A, Constante M, Walshe M, Hall M-H, Friston K, Murray R, Bramon E, 2014. Resting EEG in psychosis and at-risk populations— a possible endophenotype? Schizophr. Res. 153 (1–3), 96–102. 10.1016/j.schres.2013.12.017. 24486144 PMC3969576

[R124] Rashid B, Arbabshirani MR, Damaraju E, Cetin MS, Miller R, Pearlson GD, Calhoun VD, 2016. Classification of schizophrenia and bipolar patients using static and dynamic resting-state fMRI brain connectivity. NeuroImage 134, 645–657. 10.1016/j.neuroimage.2016.04.051. 27118088 PMC4912868

[R125] Ravan M, Noroozi A, Sanchez MM, Borden L, Alam N, Flor-Henry P, Colic S, Khodayari-Rostamabad A, Minuzzi L, Hasey G, 2024. Diagnostic deep learning algorithms that use resting EEG to distinguish major depressive disorder, bipolar disorder, and schizophrenia from each other and from healthy volunteers. J. Affect. Disord. 346, 285–298. 10.1016/j.jad.2023.11.017. 37963517

[R126] Regier DA, Narrow WE, Clarke DE, Kraemer HC, Kuramoto SJ, Kuhl EA, Kupfer DJ, 2013. DSM-5 field trials in the United States and Canada, Part II: test-retest reliability of selected categorical diagnoses. Am. J. Psychiatry 170 (1), 59–70. 10.1176/appi.ajp.2012.12070999. 23111466

[R127] Remiszewski N, Bryant JE, Rutherford SE, Marquand AF, Nelson E, Askar I, Lahti AC, Kraguljac NV, 2022. Contrasting case-control and normative reference approaches to capture clinically relevant structural brain abnormalities in patients with first-episode psychosis who are antipsychotic naive. JAMA Psychiatry 79 (11), 1133. 10.1001/jamapsychiatry.2022.3010. 36169987 PMC9520436

[R128] Ribba B, Dudal S, Lavé T, Peck RW, 2020. Model-informed artificial intelligence: reinforcement learning for precision dosing. Clin. Pharmacol. Ther. 107 (4), 853–857. 10.1002/cpt.1777. 31955414

[R129] Richens JG, Lee CM, Johri S, 2020. Improving the accuracy of medical diagnosis with causal machine learning. Nat. Commun. 11 (1), 3923. 10.1038/s41467-020-17419-7. 32782264 PMC7419549

[R130] Riecher-Rössler A, Pflueger MO, Aston J, Borgwardt SJ, Brewer WJ, Gschwandtner U, Stieglitz R-D, 2009. Efficacy of using cognitive status in predicting psychosis: a 7-year follow-up. Genotypic Neuroimaging Biomark. Schizophr. 66 (11), 1023–1030. 10.1016/j.biopsych.2009.07.020. 19733837

[R131] Saeidi M, Karwowski W, Farahani FV, Fiok K, Taiar R, Hancock PA, Al-Juaid A, 2021. Neural decoding of EEG signals with machine learning: a systematic review. Article 11 Brain Sci. 11 (11). 10.3390/brainsci11111525. PMC8615531 34827524

[R132] Santesteban-Echarri O, Paino M, Rice S, González-Blanch C, McGorry P, Gleeson J, Alvarez-Jimenez M, 2017. Predictors of functional recovery in first-episode psychosis: a systematic review and meta-analysis of longitudinal studies. Clin. Psychol. Rev. 58, 59–75. 10.1016/j.cpr.2017.09.007. 29042139

[R133] Sarpal DK, Argyelan M, Robinson DG, Szeszko PR, Karlsgodt KH, John M, Weissman N, Gallego JA, Kane JM, Lencz T, Malhotra AK, 2016. Baseline striatal functional connectivity as a predictor of response to antipsychotic drug treatment. Am. J. Psychiatry 173 (1), 69–77. 10.1176/appi.ajp.2015.14121571. 26315980 PMC4845897

[R134] Schubert KO, Clark SR, Baune BT, 2015. The use of clinical and biological characteristics to predict outcome following First Episode Psychosis. Aust. N. Z. J. Psychiatry 49 (1), 24–35. 10.1177/0004867414560650. 25430911

[R135] Shen X, Finn ES, Scheinost D, Rosenberg MD, Chun MM, Papademetris X, Constable RT, 2017. Using connectome-based predictive modeling to predict individual behavior from brain connectivity. Nat. Protoc. 12 (3), 506–518. 10.1038/nprot.2016.178. 28182017 PMC5526681

[R136] Shi W, Fan L, Wang H, Liu B, Li W, Li J, Cheng L, Chu C, Song M, Sui J, Luo N, Cui Y, Dong Z, Lu Y, Ma Y, Ma L, Li K, Chen J, Chen Y, Jiang T, 2023. Two subtypes of schizophrenia identified by an individual-level atypical pattern of tensor- based morphometric measurement. Cereb. Cortex 33 (7), 3683–3700 10.1093/cercor/bhac301. 36005854

[R137] Shim M, Hwang H-J, Kim D-W, Lee S-H, Im C-H, 2016. Machine-learning-based diagnosis of schizophrenia using combined sensor-level and source-level EEG features. Article 2–3 Schizophr. Res. 176 (2–3). 10.1016/j.schres.2016.05.007. 27427557

[R138] Simon GE, Stewart C, Yarborough BJ, Lynch F, Coleman KJ, Beck A, Operskalski BH, Penfold RB, Hunkeler EM, 2018. Mortality rates after the first diagnosis of psychotic disorder in adolescents and young adults. JAMA Psychiatry 75 (3), 254. 10.1001/jamapsychiatry.2017.4437. 29387876 PMC5885951

[R139] Solmi M, Seitidis G, Mavridis D, Correll CU, Dragioti E, Guimond S, Tuominen L, Dargél A, Carvalho AF, Fornaro M, Maes M, Monaco F, Song M, Il Shin J, Cortese S, 2023. Incidence, prevalence, and global burden of schizophrenia—data, with critical appraisal, from the Global Burden of Disease (GBD) 2019. Mol. Psychiatry. 10.1038/s41380-023-02138-4. 37500825

[R140] Spilka MJ, Raugh IM, Berglund AM, Visser KF, Strauss GP, 2023. Reinforcement learning profiles and negative symptoms across chronic and clinical high- risk phases of psychotic illness. Eur. Arch. Psychiatry Clin. Neurosci. 273 (8), 1747–1760. 10.1007/s00406-022-01528-z. 36477406

[R141] Stapleton CJ, Acharjee A, Irvine HJ, Wolcott ZC, Patel AB, Kimberly WT, 2019. High-throughput metabolite profiling: Identification of plasma taurine as a potential biomarker of functional outcome after aneurysmal subarachnoid hemorrhage. J. Neurosurg. 133 (6), 1842–1849. 10.3171/2019.9.JNS191346. 31756713

[R142] Stopper CM, Floresco SB, 2015. Dopaminergic circuitry and risk/reward decision making: implications for schizophrenia. Schizophr. Bull. 41 (1), 9–14. 10.1093/schbul/sbu165. 25406370 PMC4266315

[R143] Strauss GP, Datta R, Armstrong W, Raugh IM, Kraguljac NV, Lahti AC, 2021. Reinforcement learning abnormalities in the attenuated psychosis syndrome and first episode psychosis. Eur. Neuropsychopharmacol. 47, 11–19. 10.1016/j.euroneuro.2021.03.014. 33819817 PMC8197752

[R144] Sun Y, Zhang Z, Kakkos I, Matsopoulos GK, Yuan J, Suckling J, Xu L, Cao S, Chen W, Hu X, Li T, Sim K, Qi P, Sun Y, 2022. Inferring the individual psychopathologic deficits with structural connectivity in a longitudinal cohort of schizophrenia. IEEE J. Biomed. Health Inform. 26 (6), 2536–2546. 10.1109/JBHI.2021.3139701. 34982705

[R145] Thuné H, Recasens M, Uhlhaas PJ, 2016. The 40-Hz auditory steady-state response in patients with schizophrenia: a meta-analysis. Article 11 JAMA Psychiatry 73 (11). 10.1001/jamapsychiatry.2016.2619. 27732692

[R146] Tomoda T, Sumitomo A, Jaaro-Peled H, Sawa A, 2016. Utility and validity of DISC1 mouse models in biological psychiatry. Neuroscience 321, 99–107. 10.1016/j.neuroscience.2015.12.061. 26768401 PMC4803604

[R147] Valletta JJ, Torney C, Kings M, Thornton A, Madden J, 2017. Applications of machine learning in animal behaviour studies. Anim. Behav. 124, 203–220. 10.1016/j.anbehav.2016.12.005.

[R148] Vasudevan S, Saha A, Tarver ME, Patel B, 2022. Digital biomarkers: convergence of digital health technologies and biomarkers. Npj Digit. Med. 5 (1), 36. 10.1038/s41746-022-00583-z. 35338234 PMC8956713

[R149] Voineskos AN, Jacobs GR, Ameis SH, 2020. Neuroimaging heterogeneity in psychosis: neurobiological underpinnings and opportunities for prognostic and therapeutic innovation. Biol. Psychiatry 88 (1), 95–102. 10.1016/j.biopsych.2019.09.004. 31668548 PMC7075720

[R150] Wager S, Athey S, 2018. Estimation and inference of heterogeneous treatment effects using random forests. J. Am. Stat. Assoc. 113 (523), 1228–1242. 10.1080/01621459.2017.1319839.

[R151] Watson DS, Krutzinna J, Bruce IN, Griffiths CE, McInnes IB, Barnes MR, Floridi L, 2019. Clinical applications of machine learning algorithms: beyond the black box. BMJ l886. 10.1136/bmj.l886. 30862612

[R152] Wen Y, Zhou C, Chen L, Deng Y, Cleusix M, Jenni R, Conus P, Do KQ, Xin L, 2023. Bridging structural MRI with cognitive function for individual level classification of early psychosis via deep learning. Front. Psychiatry 13, 1075564. 10.3389/fpsyt.2022.1075564. 36704734 PMC9871589

[R153] Wendler A, Wehling M, 2010. The translatability of animal models for clinical development: biomarkers and disease models. Curr. Opin. Pharmacol. 10 (5), 601–606. 10.1016/j.coph.2010.05.009. 20542730

[R154] Whiteway MR, Biderman D, Friedman Y, Dipoppa M, Buchanan EK, Wu A, Zhou J, Bonacchi N, Miska NJ, Noel J-P, Rodriguez E, Schartner M, Socha K, Urai AE, Salzman CD, Laboratory TIB, Cunningham JP, Paninski L, 2021. Partitioning variability in animal behavioral videos using semi-supervised variational autoencoders. PLOS Comput. Biol. 17 (9), e1009439 10.1371/journal.pcbi.1009439. 34550974 PMC8489729

[R155] Wiltschko AB, Tsukahara T, Zeine A, Anyoha R, Gillis WF, Markowitz JE, Peterson RE, Katon J, Johnson MJ, Datta SR, 2020. Revealing the structure of pharmacobehavioral space through motion sequencing. Article 11 Nat. Neurosci. 23 (11). 10.1038/s41593-020-00706-3. PMC7606807 32958923

[R156] Wolfers T, Doan NT, Kaufmann T, Alnæs D, Moberget T, Agartz I, Buitelaar JK, Ueland T, Melle I, Franke B, Andreassen OA, Beckmann CF, Westlye LT, Marquand AF, 2018. Mapping the heterogeneous phenotype of schizophrenia and bipolar disorder using normative models. JAMA Psychiatry 75 (11), 1146. 10.1001/jamapsychiatry.2018.2467. 30304337 PMC6248110

[R157] Wu C-S, Luedtke AR, Sadikova E, Tsai H-J, Liao S-C, Liu C-C, Gau SS-F, VanderWeele TJ, Kessler RC, 2020. Development and validation of a machine learning individualized treatment rule in first-episode schizophrenia. JAMA Netw. Open 3 (2), e1921660. 10.1001/jamanetworkopen.2019.21660. 32083693 PMC7043195

[R158] Wu EQ, Shi L, Birnbaum H, Hudson T, Kessler R, 2006. Annual prevalence of diagnosed schizophrenia in the USA: a claims data analysis approach. Psychol. Med. 36 (11), 1535–1540. 10.1017/S0033291706008191. 16907994

[R159] Xiao Y, Liao W, Long Z, Tao B, Zhao Q, Luo C, Tamminga CA, Keshavan MS, Pearlson GD, Clementz BA, Gershon ES, Ivleva EI, Keedy SK, Biswal BB, Mechelli A, Lencer R, Sweeney JA, Lui S, Gong Q, 2022. Subtyping schizophrenia patients based on patterns of structural brain alterations. Schizophr. Bull. 48 (1), 241–250. 10.1093/schbul/sbab110. 34508358 PMC8781382

[R160] Padmanabha Reddy Y.C.a, Pulabaigari V, E B, 2018. Semi-supervised learning: a brief review. Int. J. Eng. Technol. 7, 81. 10.14419/ijet.v7i1.8.9977.

[R161] Yang Z, Lee SH, Abdul Rashid NA, See YM, Dauwels J, Tan BL, Lee J, 2021. Predicting real-world functioning in schizophrenia: the relative contributions of neurocognition, functional capacity, and negative symptoms. Front. Psychiatry 12, 639536. 10.3389/fpsyt.2021.639536. 33815171 PMC8017150

[R162] Yassin W, Nakatani H, Zhu Y, Kojima M, Owada K, Kuwabara H, Gonoi W, Aoki Y, Takao H, Natsubori T, Iwashiro N, Kasai K, Kano Y, Abe O, Yamasue H, Koike S, 2020. Machine-learning classification using neuroimaging data in schizophrenia, autism, ultra-high risk and first-episode psychosis. Transl. Psychiatry 10 (1), 278. 10.1038/s41398-020-00965-5. 32801298 PMC7429957

[R163] Young AL, Marinescu RV, Oxtoby NP, Bocchetta M, Yong K, Firth NC, Cash DM, Thomas DL, Dick KM, Cardoso J, Van Swieten J, Borroni B, Galimberti D, Masellis M, Tartaglia MC, Rowe JB, Graff C, Tagliavini F, Frisoni GB, Furst AJ, 2018. Uncovering the heterogeneity and temporal complexity of neurodegenerative diseases with Subtype and Stage Inference. Nat. Commun. 9 (1), 4273. 10.1038/s41467-018-05892-0. 30323170 PMC6189176

[R164] Zarate D, Stavropoulos V, Ball M, De Sena Collier G, Jacobson NC, 2022. Correction: exploring the digital footprint of depression: a PRISMA systematic literature review of the empirical evidence. BMC Psychiatry 22 (1), 530. 10.1186/s12888-022-04153-1. 35733121 PMC9214685

[R165] Zhang J, Zhang R, Peng Y, Aa J, Wang G, 2023. AI machine learning technique characterizes potential markers of depression in two animal models of depression. Brain Sci. 13(5), Artic. 5. 10.3390/brainsci13050763. PMC10216178 37239235

[R166] Zhang W, Sweeney JA, Bishop JR, Gong Q, Lui S, 2023. Biological subtyping of psychiatric syndromes as a pathway for advances in drug discovery and personalized medicine. Nat. Ment. Health 1 (2), 88–99 10.1038/s44220-023-00019-x.

[R167] Zhang X, Yu H, Bai R, Ma C, 2020. Identification and characterization of biomarkers and their role in opioid addiction by integrated bioinformatics analysis. Front. Neurosci. 14 https://www.frontiersin.org/journals/neuroscience/articles/10.3389/fnins.2020.608349. 10.3389/fnins.2020.608349 PMC7729193 33328875

[R168] Zheng P, Zeng B, Liu M, Chen J, Pan J, Han Y, Liu Y, Cheng K, Zhou C, Wang H, Zhou X, Gui S, Perry SW, Wong M-L, Licinio J, Wei H, Xie P, 2019. The gut microbiome from patients with schizophrenia modulates the glutamate-glutamine- GABA cycle and schizophrenia-relevant behaviors in mice. eaau8317 Sci. Adv. 5 (2). 10.1126/sciadv.aau8317. 30775438 PMC6365110

[R169] Zheng W, Zhang Q-E, Cai D-B, Ng CH, Ungvari GS, Ning Y-P, Xiang Y-T, 2018. Neurocognitive dysfunction in subjects at clinical high risk for psychosis: a meta- analysis. J. Psychiatr. Res. 103, 38–45. 10.1016/j.jpsychires.2018.05.001. 29772485

[R170] Zhou T-H, Mueller NE, Spencer KM, Mallya SG, Lewandowski KE, Norris LA, Levy DL, Cohen BM, Öngür D, Hall M-H, 2018. Auditory steady state response deficits are associated with symptom severity and poor functioning in patients with psychotic disorder. Schizophr. Res. 201, 278–286. 10.1016/j.schres.2018.05.027. 29807805 PMC7003536

[R171] Zhu X, Wang C, Yu J, Weng J, Han B, Liu Y, Tang X, Pan B, 2023. Identification of immune-related biomarkers in peripheral blood of schizophrenia using bioinformatic methods and machine learning algorithms. Front. Cell. Neurosci. 17, 1256184. 10.3389/fncel.2023.1256184. 37841288 PMC10568181

[R172] Zhu Y, Maikusa N, Radua J, Sämann P, Fusar-Poli P, 2023. Using brain structural neuroimaging measures to predict psychosis onset for individuals at clinical high-risk [Preprint]. Review. 10.21203/rs.3.rs-3267539/v1. PMC11189817 38332374

[R173] Zhu Y, Nakatani H, Yassin W, Maikusa N, Okada N, Kunimatsu A, Abe O, Kuwabara H, Yamasue H, Kasai K, Okanoya K, Koike S, 2022. Application of a machine learning algorithm for structural brain images in chronic schizophrenia to earlier clinical stages of psychosis and autism spectrum disorder: a multiprotocol imaging dataset study. Schizophr. Bull. 48 (3), 563–574. 10.1093/schbul/sbac030. 35352811 PMC9077435

[R174] Ziermans T, Wit S, de Schothorst P, Sprong M, Engeland H, van, Kahn R, Durston S, 2014. Neurocognitive and clinical predictors of long-term outcome in adolescents at ultra-high risk for psychosis: a 6-year follow-up. PLoS One 9 (4), e93994. 10.1371/journal.pone.0093994. 24705808 PMC3976376

